# Population structure, adaptation and divergence of the meadow spittlebug, *Philaenus spumarius* (Hemiptera, Aphrophoridae), revealed by genomic and morphological data

**DOI:** 10.7717/peerj.11425

**Published:** 2021-06-01

**Authors:** Sofia G. Seabra, Ana S.B. Rodrigues, Sara E. Silva, Ana Carina Neto, Francisco Pina-Martins, Eduardo Marabuto, Vinton Thompson, Michael R. Wilson, Selçuk Yurtsever, Antti Halkka, Maria Teresa Rebelo, Paulo A.V. Borges, José A. Quartau, Chris D. Jiggins, Octávio S. Paulo

**Affiliations:** 1 E3c - Centre for Ecology, Evolution and Environmental Changes, Departamento de Biologia Animal, Faculdade de Ciências, Universidade de Lisboa, Lisboa, Portugal; 2 CESAM - Centre for Environmental and Marine Studies, Departamento de Biologia Animal, Faculdade de Ciências, Universidade de Lisboa, Lisboa, Portugal; 3 American Museum of Natural History, New York, USA; 4 National Museum of Wales, Department of Natural Sciences, Cardiff, United Kingdom; 5 Trakya University, Biology Department, Science Faculty, Edirne, Turkey; 6 Department of Biological and Environmental Sciences, University of Helsinki, Helsinki, Finland; 7 cE3c - Centre for Ecology, Evolution and Environmental Changes/Azorean Biodiversity Group, Faculty of Agriculture and Environment, Department of Environmental Sciences and Engineering, Universidade dos Açores, Angra do Heroísmo, Açores, Portugal; 8 Department of Zoology, University of Cambridge, Cambridge, United Kingdom

**Keywords:** Population genomics, RAD sequencing, Aedeagus, Species divergence, Local adaptation, *Philaenus spumarius*

## Abstract

Understanding patterns of population differentiation and gene flow in insect vectors of plant diseases is crucial for the implementation of management programs of disease. We investigated morphological and genome-wide variation across the distribution range of the spittlebug *Philaenus spumarius* (Linnaeus, 1758) (Hemiptera, Auchenorrhyncha, Aphrophoridae), presently the most important vector of the plant pathogenic bacterium *Xylella fastidiosa* Wells et al., 1987 in Europe. We found genome-wide divergence between *P. spumarius* and a very closely related species, *P. tesselatus* Melichar, 1899, at RAD sequencing markers. The two species may be identified by the morphology of male genitalia but are not differentiated at mitochondrial COI, making DNA barcoding with this gene ineffective. This highlights the importance of using integrative approaches in taxonomy. We detected admixture between *P. tesselatus* from Morocco and *P. spumarius* from the Iberian Peninsula, suggesting gene-flow between them. Within *P. spumarius*, we found a pattern of isolation-by-distance in European populations, likely acting alongside other factors restricting gene flow. Varying levels of co-occurrence of different lineages, showing heterogeneous levels of admixture, suggest other isolation mechanisms. The transatlantic populations of North America and Azores were genetically closer to the British population analyzed here, suggesting an origin from North-Western Europe, as already detected with mitochondrial DNA. Nevertheless, these may have been produced through different colonization events. We detected SNPs with signatures of positive selection associated with environmental variables, especially related to extremes and range variation in temperature and precipitation. The population genomics approach provided new insights into the patterns of divergence, gene flow and adaptation in these spittlebugs and led to several hypotheses that require further local investigation.

## Introduction

Speciation involves the evolution of reproductive isolation and the buildup of genetic differentiation through selection and drift, but gene flow can counteract such divergence by homogenizing allelic variation and also by allowing recombination to oppose or break associations between loci underlying isolating traits (Smadja & Butlin, 2011; Sousa & Hey, 2013). However, several mechanisms may favour divergence in the face of gene flow, such as ecologically driven selection or sexual selection (Smadja & Butlin, 2011; Nosil, 2012). According to the genic model of speciation, at the start of the speciation process, and in the presence of gene flow, a few localized regions in the genome subject to divergent selection will differentiate, while the remaining genome continues to be freely exchanged between populations (Wu, 2001). Genome-wide analyses have allowed the detection of these “genomic islands” of differentiation in several systems (e.g., Malinsky et al., 2015; Vijay et al., 2016), although other processes not related to speciation or reproductive isolation may also be responsible for them, such as linked selection, variable recombination rates and/or density of targets of selection (Wolf & Ellegren, 2017). As populations diverge through the action of selection and drift, a genome-wide differentiation will emerge and eventually lead to full reproductive isolation and diversification. Designated species may thus lie somewhere in this “speciation continuum”, with different levels of divergence and gene flow (Hendry et al., 2009; Peccoud et al., 2009; Renaut et al., 2012; Riesch et al., 2017).

Distinguishing taxa and understanding the patterns of gene flow and local adaptation in insect species that transmit diseases are crucial for better management of those diseases (Busvine, 1980; Pélissié et al., 2018; Bahrndorff et al., 2020). *Philaenus spumarius* (Linnaeus, 1758) (Insecta, Hemiptera, Auchenorrhyncha, Aphrophoridae), the meadow spittlebug, is a xylem-feeding vector of *Xylella fastidiosa* Wells et al., 1987, a plant pathogenic bacterium of South American origin that is emergent in Europe (Saponari et al., 2014). Olive quick decline syndrome (OQDS), caused by *X. fastidiosa*, was first detected in Apulia, southern Italy in 2013, where it soon became clear that *P. spumarius* was the most important vector (Saponari et al., 2014; Cornara et al., 2017). Since then, *X. fastidiosa* has been detected in several other European countries and is a cause for major concern (EFSA et al , 2019). *X. fastidiosa* is native to the Americas, where it causes important diseases such as Pierce’s disease of grapevine, citrus variegated chlorosis, almond leaf scorch and several others in perennial crops and ornamental plants (Baldi & La Porta, 2017). There, the main vectors are sharpshooters (another xylem-feeding Auchenorrhyncha group, the Cicadellidae, Cicadellinae), while spittlebugs appear to have a small, but perhaps not negligible, epidemiological importance (Almeida et al., 2019; Cornara et al., 2019; Beal et al., 2021). One of the main vectors of Pierce’s disease of grapevines in California is the glassy-winged sharpshooter *Homalodisca vitripennis* (Germar, 1821). It is native to the southern United States and it became established in late 1990’s in California, being a costly invasive species to agriculture. Population genetic structure studies based on DNA fingerprinting and mitochondrial DNA on this species have revealed highly differentiated geographic groups in the natural range and indicated that the likely sources of the California insects were in Texas (León, Jones & Morgan, 2004; Smith, 2005). This inspired further work in Texas, which led to a better understanding of the natural population dynamics (Yoon et al., 2014). This demonstrates the potential importance of knowledge of the population genetic structure of *P. spumarius* for understanding the dynamics of the spread of *X. fastidiosa* in Europe. Since the vectors are the only means of natural dissemination of *X. fastidiosa* (Sicard et al., 2018), this information is crucial for the successful management of this pathogen and should be included in models of risk assessment (EFSA Panel on Plant Health , 2015).

*P. spumarius* is a polyphagous xylem-feeding insect, widespread in the Holarctic, whose nymphs produce a protective foam (spittle masses) from their liquid excretion. Humidity and temperature are particularly limiting in the earlier nymphal stages (Weaver & King, 1954). In general, adults live during one reproductive season in spring/summer, and then at the end of summer/autumn the females oviposit and the eggs overwinter in the vegetation until they hatch in the following spring/summer (Halkka & Halkka, 1990). This species is thought to have a Palaearctic origin, and to have recently colonized North America, the Azorean islands, Hawaii and New Zealand. These introductions were likely mediated by humans (Halkka & Halkka, 1990; Rodrigues et al., 2014), although natural colonization cannot be excluded for the S. Miguel island in the Azores (Borges et al., 2018; Rodrigues et al., 2014) as the populations in this island are restricted to high elevation native vegetation of the oriental and geologically oldest part of this island. In parts of North America it has been a crop pest (Weaver & King, 1954), but surveys of the spittle masses along coastal California have revealed a recent population decline of this species, very accentuated in some places (Karban & Strauss, 2004; Karban & Huntzinger, 2018). The population in the Wonalancet, New Hampshire, site sampled for this report in 2010 has also markedly declined since that date (V. Thompson, 2020, unpublished data).

Previous studies based on mitochondrial and nuclear DNA genes have revealed the major phylogeographic patterns in *P. spumarius* (Maryańska-Nadachowska, Kajtoch & Lachowska, 2011; Rodrigues et al., 2014). Two main mitochondrial lineages have initially diverged during the Pleistocene: the “Western”, currently found in the Mediterranean region and also in Central and Northern Europe, and the “North-Eastern”, currently found from Eastern Asia to Central and Northern Europe. The “Western” lineage is further differentiated into sublineages: the “Western”, predominant in the Iberian Peninsula but also in western parts of Central and Northern Europe; and the “Eastern-Mediterranean” present in the Balkans and Middle East and others around Black Sea and in the Caucasus (Maryańska-Nadachowska et al., 2010; Rodrigues et al., 2014). These lineages also co-occur in several contact zones (Lis et al., 2014; Rodrigues et al., 2014). The occurrence of different infection rates of the maternally inherited endosymbiont *Wolbachia* in the different lineages of *P. spumarius* has pointed to a possible mechanism to explain the maintenance of genetic differentiation in the Carpathians contact zone (Lis, Maryańska-Nadachowska & Kajtoch , 2015). Previous studies have also revealed close relationships and even shared haplotypes between samples from North America, Azores, New Zealand and those from Great Britain (Rodrigues et al., 2014), indicating a recent human-assisted colonization, as previously suggested for North America and New Zealand (Hamilton, 1979; Yurtsever, 2002).

Seven other species of the genus *Philaenus* occur in the Mediterranean area, having much more restricted distribution ranges which partially overlap that of *P. spumarius* (Drosopoulos, 2003; Maryańska-Nadachowska et al., 2010). One such species is *Philaenus tesselatus* Melichar, 1889, which was originally described from Tunisia and was later synonymized with *P. spumarius* (Nast, 1972), being considered a geographic subspecies. Later, the synonymy was re-assessed based on morphological evaluation, with the best diagnostic characters being the size and shape of the appendages of the male aedeagus (Drosopoulos & Quartau, 2002). However, geographic variation in the curvature of the aedeagal apical appendages in *P. spumarius* has been reported in both Europe and North America (Wagner, 1955; Wagner, 1959; Hamilton, 1979). Such variation in aedeagus structure within *P. tesselatus* is still largely unexplored (Drosopoulos & Quartau, 2002). Recent genetics studies based on mitochondrial *cytochrome c oxidase I* (COI) and *cytochrome b* (cytB), as well as on nuclear *internal transcribed spacer 2* (ITS2) and *elongation factor 1-alpha* (EF-1alpha) DNA sequence analysis have questioned the species status of *P. tesselatus*, since individuals showing *P. tesselatus*-like male genitalia have the same or very similar sequences to *P. spumarius* (Maryańska-Nadachowska, Kajtoch & Lachowska, 2011; Rodrigues et al., 2014). It is expected that genome-wide markers will provide greater resolution to understand the divergence between these cryptic species. Delimitation of species boundaries is a difficult taxonomic endeavour but it is now widely recognised that an integrative taxonomic approach should include phenotypic, genetic (with a large number of nuclear and mitochondrial markers), and ecological data (Edwards & Knowles, 2014; Tonzo, Papadopoulou & Ortego, 2019).

In this study, we applied restriction site-associated DNA sequencing (RAD-seq), a reduced-representation sequencing approach that simultaneously discovers and genotypes thousands of single nucleotide polymorphisms for a large number of individuals (Baird et al., 2008; Andrews et al., 2016). We had three main objectives: (i) to characterize the morphological (appendages of male aedeagus) and genome-wide divergence between *P. spumarius* and *P. tesselatus;* (ii) characterise the patterns of genome-wide differentiation of *P. spumarius* populations across the distribution range of the species; and (iii) detect local adaptation by finding genomic regions under selection and associated with environmental variation. The information on gene flow between populations and on the environmental factors associated with local adaptation, as well as on the most appropriate diagnostic methods for the identification of the closely related *P. spumarius* and *P. tesselatus,* will be important for future risk assessment of *X. fastidiosa* spread in Europe.

## Materials & Methods

### Sampling

**Figure 1:**
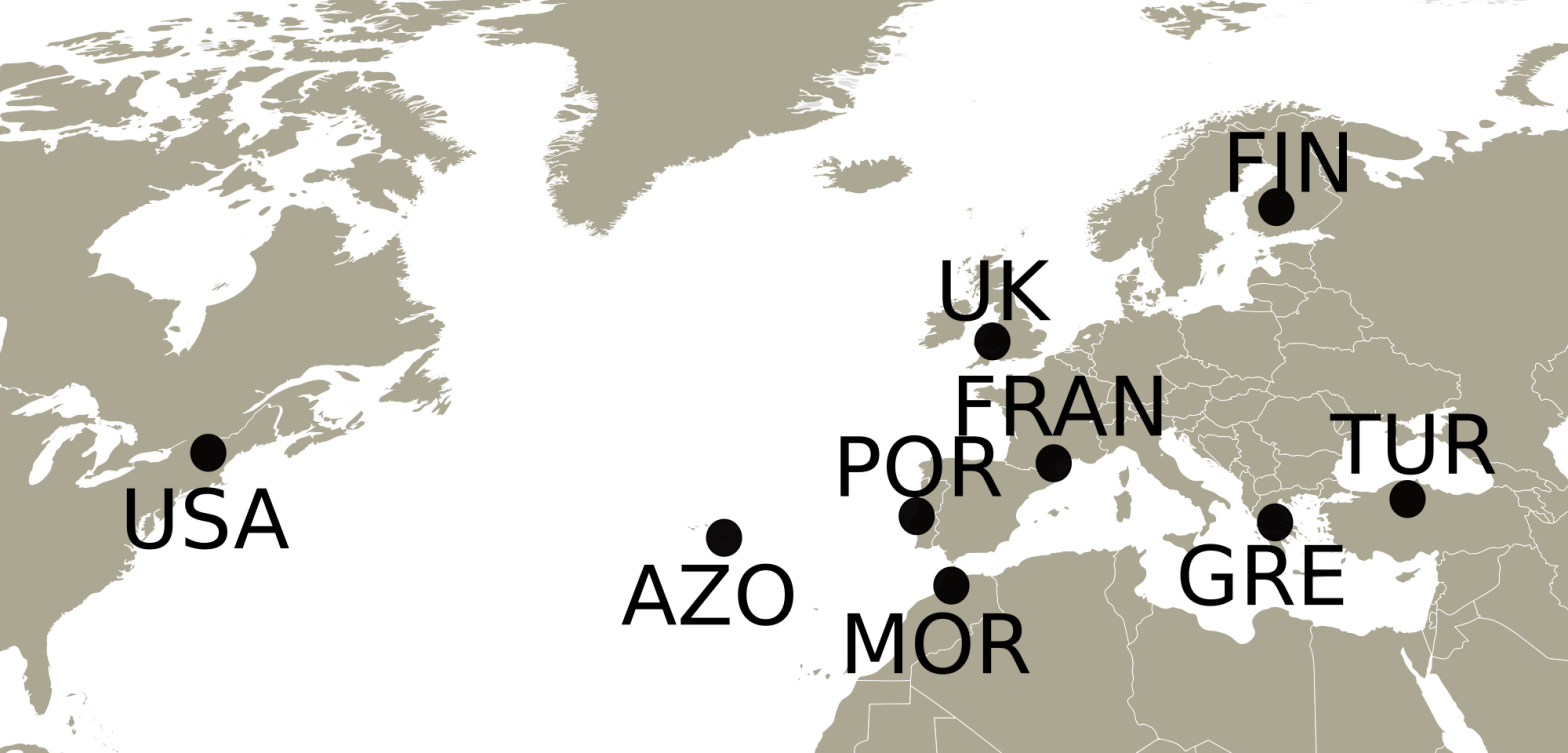
Map with the sampling locations of *Philaenus*. The points indicate the sampling locations of *Philaenus spumarius* in Turkey (TUR), Greece (GRE), Finland (FIN), France (FRAN), Portugal (POR), United Kingdom (UK), Azores (AZO) and United States of America (USA), and of *Philaenus tesselatus* in Morocco (MOR). In Morocco, three locations were sampled (details in Table S1).

Adults and nymphs of *Philaenus spumarius* were collected in 2010 and 2011 from eight populations (Fig. 1) across the distribution range of the species: Cerkes, Anatolia, Turkey (TUR); Mount Parnassus, Greece (GRE); Haapamäki-Keuruu, Finland (FIN); Fitou, South of France (FRAN); Gouveia/Fontanelas, Sintra, Portugal (POR); Aberdare, South Wales, United Kingdom (UK); S. Miguel island, Azores (AZO); Wonalancet, New Hampshire, United States of America (USA). *P. tesselatus* was sampled in Morocco (MOR), from three main localities: near Azrou, near Rabat and near Ceuta. In total, 170 specimens were sequenced, including 20–22 individuals from each sampling site except Morocco, from where only 7 specimens were included (Table S1). Since sampling in this last location was initially intended for a phylogeographic characterization (Rodrigues et al., 2014), only a few individuals were collected from each site.

Nymphs were hand collected from the spittle masses they produce. Adults were collected by sweeping the vegetation with an entomological net. Both nymphs and adults occur and feed on large numbers of host plant species and no particular hosts were selected during sweeping and hand collection. Efforts by others to show associations between common hosts and the genetically determined color polymorphism gave negative results (Halkka et al., 1967) and we know of no evidence suggesting host-specific genetic differentiation.

Insects were preserved in absolute or 96% ethanol until they were subjected to DNA extraction after up to one year.

### Morphological characters

*Philaenus* species distinction based on the morphology of male genitalia was accomplished for a subset of 38 males from across all populations, except the AZO and FRAN, for which only immature individuals or females were collected. Nine additional males from MOR, POR and TUR were included to increase morphological sample size, but were not used for genetic analyses (Table S1). Preparation and measurements of male genitalia were done as detailed in  Supplementary Information 1.

**Figure 2:**
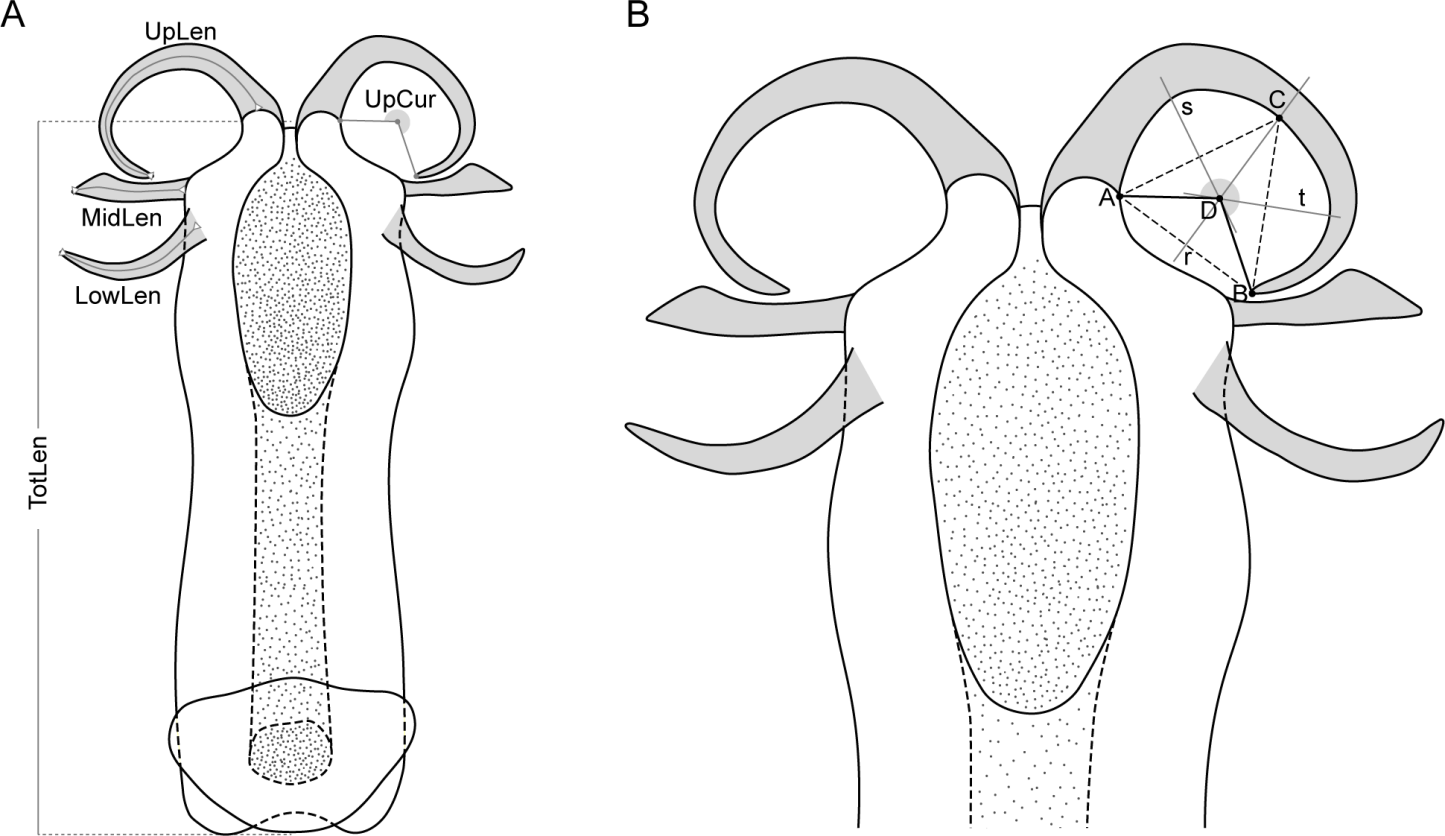
Schematic representation of the aedeagus of *Philaenus* with morphometric characteres measured. (A) Morphometric characters measured on the aedeagus of *Philaenus* spittlebugs: *TotLen –* total length of aedeagus; *LowLen*, length of lower appendages (left and right); *MidLen*, length of middle appendages (left and right); *UpLen*, length of upper appendages (left and right); *UpCur*, curvature of upper appendages (left and right). (B) Diagram of geometric measurements of the curvature of the aedeagus upper appendages.

Five variables calculated from the nine measurements (Fig. 2) were used in the morphometric analysis: total length of aedeagus (TotLen), mean length of lower appendages (LowLen), mean length of middle appendages (MidLen), mean length of upper appendages (UpLen), mean curvature of upper appendages (UpCur). The mean value of measurements of paired structures was considered instead of both left and right measurements individually to reduce some of the variability and the number of specimens to be dropped out of the analysis due to missing values related to appendages that were occasionally broken or tilted during aedeagus removal.

A Principal Component Analysis (PCA) was used to evaluate if morphological characters of the aedeagus could separate *Philaenus* species and/or populations. PCA was applied to standardized variables (centred by the mean and scaled by the variance), since they were measured in different units. Three specimens were left out of the analysis due to missing values. The analysis was performed in R version 3.4.1 (R Core Team, 2017) using the prcomp function and figures were produced using the package ggplot2 version 3.2.1 (Wickham, 2016).

### DNA extraction and mitochondrial DNA analyses

DNA was extracted from the head and thorax of each specimen using the DNeasy Blood & Tissue kit (Qiagen) following the manufacturer’s instructions and including an RNase A treatment step. Wings and abdomen were not used for DNA extraction to avoid extracting DNA of endosymbionts and parasites. The obtained DNA was assessed for the presence of a high molecular weight band on the agarose gel after electrophoresis, and it was quantified in Qubit 2.0 (Invitrogen), using Qubit dsDNA HS Assay kit.

A subset of 48 specimens from the nine areas were sequenced for mitochondrial DNA (Table S1) from which we amplified an 800 bp fragment of the 3′-end of the mitochondrial gene cytochrome *c* oxidase subunit I (COI) by polymerase chain reaction (PCR). Primers used were: C1-J-2195 (5′–TTGATTTTTTGGTCATCCAGAAGT–3′) and TL2-N-3014 (5′–TCCAATGCACTAATCTGCCATATTA–3′) (Simon et al., 1994). PCR was performed in a 12.5 μL reaction volume containing: 1×Colorless GoTaq Flexi Buffer, 2 mM MgCl_2_, 0.2 mM dNTPs, 0.6 mg/mL of BSA, 0.5 μM of each primer, 0.0375 U GoTaq DNA Polimerase (Promega) and approximately 30 ng of DNA. PCR conditions were: an initial denaturation step at 95 °C for 5 min, followed by 35 cycles of denaturation at 95 °C for 45 s, annealing at 50 °C for 35 s and extension at 72 °C for 2 min, with a final extension period at 72 °C for 10 min.

Chromatograms were verified and edited using SEQUENCHER v. 4.0.5 (Gene Codes Corporation), they were aligned using CLUSTAL W on BIOEDIT v. 7.0.9 (Thompson, Higgins & Gibson, 1994; Hall, 1999) and subsequently trimmed to the same length. We followed the designation of haplotypes of Rodrigues et al. (2014). A median-joining haplotype network was constructed using POPART version 1.7 (Bandelt, Forster & Rohl, 1999; Leigh & Bryant, 2015).

### RAD libraries preparation and sequencing

RAD libraries were prepared using a protocol by Etter et al. (2011), with modifications as in Rodrigues et al. (2016). The restriction enzyme used was *SbfI* (New England Biolabs). Six libraries were prepared, with 28 to 31 individually barcoded samples multiplexed. The libraries were sequenced on three lanes of an Illumina HiSeq 2000 in paired-end mode (2 × 100 bp) at Genepool (Ashworth Laboratories) (http://genepool.bio.ed.ac.uk/). The individuals from each population were distributed over the different libraries and lanes to avoid library or lane-specific biases.

### Assembly and SNP calling

The sequence reads from each run were examined by *process_radtags* from STACKS version 1.45 (Catchen et al., 2013), to remove those with uncalled bases and low-quality scores (phred score lower than 10), to check that the barcode and restriction site were intact in each read and to demultiplex the samples based on the barcode identification. Reads were trimmed at the 3′end, using TRIMMOMATIC v. 0.38, to keep only 87 bases, since preliminary analyses using the entire read revealed a high number of (possibly false) SNPs at the 3′end after this number of bases (data not shown). This may be due to higher sequencing errors towards the end of the reads characteristic of Illumina sequencing (Dohm et al., 2008). The trimmed reads were de novo assembled into “stacks” (identical sets of reads, called loci) for each individual using the STACKS module *ustacks*. The minimum depth of coverage to build a stack (-m) was set to 10, the maximum number of nucleotide differences allowed between stacks to form a locus (-M) was set to 2. Then the STACKS module *cstacks* was used to build a catalog by merging stacks (loci) from multiple individuals, using the default options. The module *sstacks* was used to match loci from an individual against the catalog. Stacks with very high coverage were removed since they may represent highly repetitive regions and that may include non-orthologous sequences.

Finally, the *populations* module was used to create a Variant Call Format (VCF) file with the bi-allelic genotypes of each individual for each variable nucleotide position. The minimum number of populations a locus must be present in to process a locus (-p) was set to the number of populations analyzed (eight or nine, excluding or including Morocco, respectively–see below), the minimum percentage of individuals in a population required to process a locus for that population (-r) was set to 0.5 (50%). Only one SNP per locus was kept, using the option –write_random_snp. Other parameters (-M 2, 3 and 6, -m 5 and 10, -n 1 and 4, - -min_maf 0.05 or with no min maf) were tested and the differences were assessed by general patterns in the Principal Component Analysis.

The VCF file was then filtered using VCFTOOLS (v 0.1.14) (Danecek et al., 2011), excluding sites with less than 75% of individuals with genotype (–max-missing 0.75) and/or with minor allele count of 2 (–mac 2), to exclude singletons (Linck & Battey, 2019). In order to exclude overclustered loci, we filtered out those with a mean depth (across individuals) higher than 200×(–max-meanDP 200).

The filtered VCF file with the SNP genotypes was converted into the file formats needed for the different analysis programs using PGDSPIDER 2.0.4.0 (Lischer & Excoffier, 2012). For conversion of GESTE format to BAYPASS format, we used the script https://github.com/CoBiG2/RAD_Tools/blob/master/geste2baypass.py as of commit b99636e.

VCFTOOLS was used to calculate summary statistics of coverage and percentage of missing data. GENETIX v. 4.05.2 was used to obtain expected and observed heterozygosity, as well as F_IS_ in each population. Pairwise differentiation between populations (F_ST_) were calculated in ARLEQUIN 3.5.1.3, and the significance of F_ST_ was obtained from permutation tests with 10,000 repetitions. Mantel tests between F_ST_/(1-F_ST_) matrices and the natural logarithm of the geographical distance (Rousset, 1997) were performed with ape package version 5.0 (http://ape-package.ird.fr/) in R version 3.4.0, using 9,999 permutations.

### Population structure

Principal Components Analysis (PCA) was used as an exploratory tool of the population structure (Novembre & Stephens, 2008). Computations of PCA were performed in R using package SNPRelate version 1.12.0 (Zheng et al., 2012). Population structure was further examined using the model-based clustering algorithm implemented in STRUCTURE v. 2.3.4 (Falush, Stephens & Pritchard, 2003; Pritchard, Stephens & Donnelly, 2000). We obtained the coefficients of ancestry using the admixture model and assuming correlated allele frequencies among populations, and K from 1 to 9, with 10 replicate runs of each, applying 50,000 steps of burnin and 1,000,000 MCMC steps after burnin. STRUCUTRE_THREADER version 1.2.2 (Pina-Martins et al., 2017) was used to parallelize the runs and to find the K best explaining the data by calculating Delta K on STRUCTURE HARVESTER (Evanno, Regnaut & Goudet, 2005; Earl & vonHoldt, 2012). CLUMPP version 1.1.2 (Jakobsson & Rosenberg, 2007) was then used to obtain the optimal alignment of ancestry proportions, by permuting the 10 replicate runs of STRUCTURE for each value of K.

The complete dataset consisted of 9 populations and 133 individuals. We also analyzed a dataset excluding *P. tesselatus* individuals from Morocco, which consisted of 8 populations and 127 individuals of *P. spumarius*. In order to compare morphological variation and genetic variation in *P. spumarius,* we used a dataset of the 32 individuals for which we had data for both morphometry and RAD-seq. We performed PCA for both types of data, as above, and we calculated Spearman correlations between the Principal Component scores obtained from both PCAs using R.

### Detection of selection–outlier analyses and environmental associations

In order to detect loci with signs of selection for the *P. spumarius* RAD-seq dataset (without Morocco), two approaches were taken: one that detects outlier loci departing from expectation under neutral demographic models (Foll & Gaggiotti, 2008; Vitalis et al., 2014), and another that detects loci associated with environmental variation between populations (Coop et al., 2010; Gautier, 2015).

Outlier analyses were carried out using BAYESCAN v. 2.1 (Foll & Gaggiotti, 2008) and SELESTIM v1.1.4 (Vitalis et al., 2014). BAYESCAN uses a Bayesian approach to estimate the posterior probability of two alternative models for each locus, with or without selection. Posterior odds are then obtained and False Discovery Rate calculated to control for multiple testing. The parameters of the chain and of the model were set to the default values. Outlier SNPs were defined to be those with *q*-values lower than 5%. SELESTIM v1.1.4 (Vitalis et al., 2014) estimates the intensity of selection at each locus and the posterior distributions of the locus-specific coefficients of selection are compared with a distribution derived from the genome-wide effect of selection using Kullback-Leibler divergence (KLD). KLD is calibrated with simulations from posterior predictive distribution based on observed data (Vitalis et al., 2014). A total of 50 pilot runs of length 1,000 were followed by a run of 1,000,000 with burnin of 10,000. The criterion for a candidate SNP for selection was defined to be the 99% quantile of the KLD distribution.

Environmental and geospatial variables used in the association analysis included 19 bioclimatic variables, as well as longitude and latitude. Bioclimatic variables were mined from WorldClim version 1.4 (release 3) (http://www.worldclim.org/) and the data was extracted for each location using DIVA-GIS 7.5.0 (http://www.diva-gis.org). Associations between SNP allele frequency differences and the environmental variables were assessed with BAYPASS v. 2.1 (Gautier, 2015), using the script Baypass_workflow.R (https://gitlab.com/StuntsPT/pyRona/blob/master/pyRona/R/Baypass_workflow.R) as of pyRONA v0.3.7 (Pina-Martins et al., 2017). Significant associations were assessed with Bayes Factor (BF) obtained with the auxiliary covariate model, considering a threshold for BF of 15. We did not exclude any variable at the start of the study based on their correlations, but we did reassess correlation between significantly associated variables. Spearman correlations between variables were calculated using R.

After finding the candidate SNPs for selection and environmental association, two datasets were created: a “neutral” dataset, for which we excluded the candidate SNPs, and a “candidate” dataset, that contained only the candidate SNPs. STRUCTURE analyses were also performed on these two datasets.

RAD tags with candidate SNPs were queried against the available *P. spumarius* partial draft genome and transcriptome (Rodrigues et al., 2016), using blastn with an e-value threshold of 1E–30. We obtained a longer sequence (100 bp extended from each end of the RAD tag) from the genome alignment, which was then queried against the NCBI nucleotide database (nr/nt) using BLASTN version 2.9.0 (Altschul et al., 1997), setting a threshold e-value of 1E–5.

Scripts used in these analyses are available at https://github.com/seabrasg/popgenom_Philaenus.git.

## Results

### Morphology of male aedeagus

**Figure 3:**
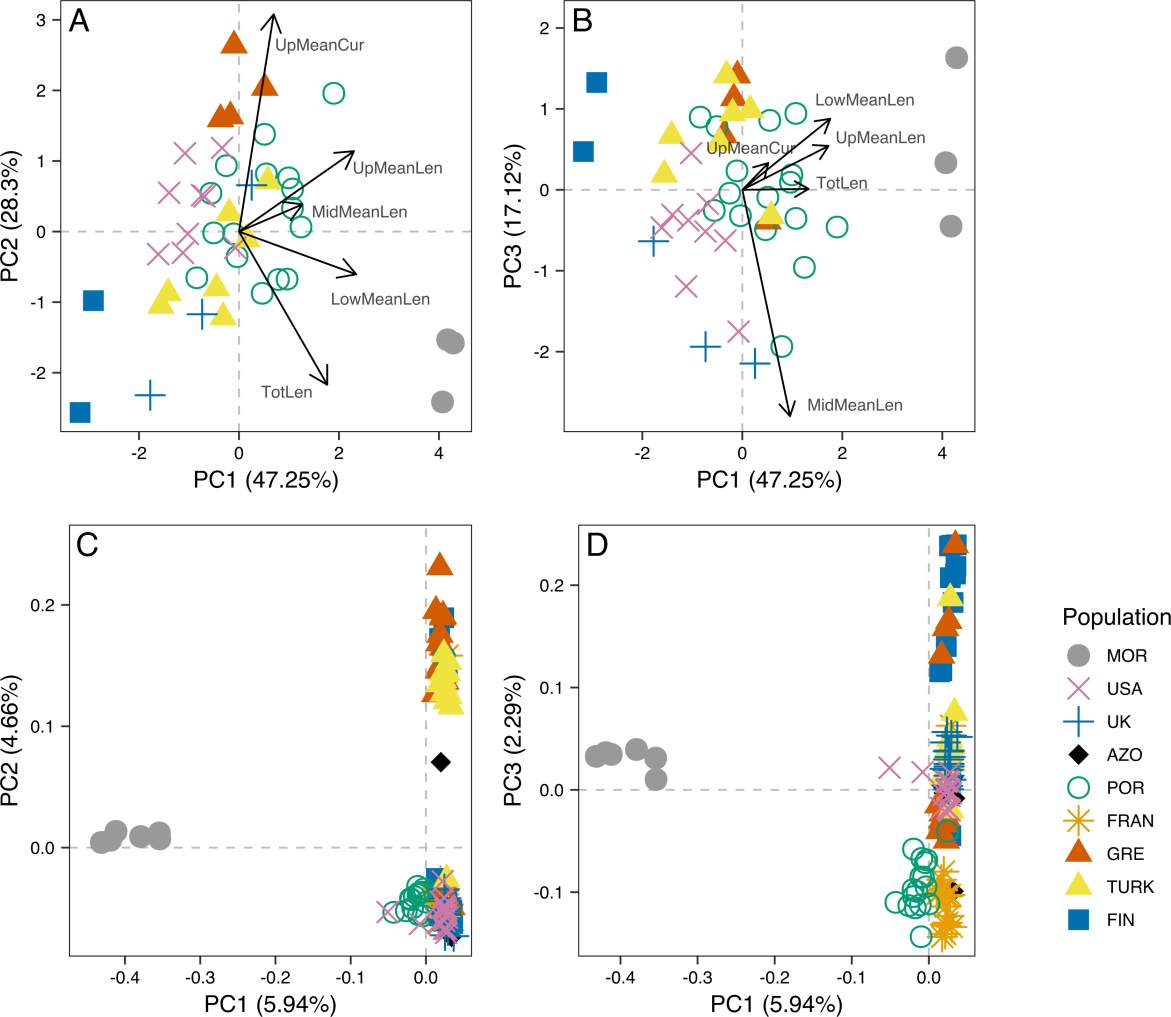
Principal component analysis of morphometric data of male aedeagus and of RAD-seq data. Scatterplots of the three first principal components (PC1, PC2 nd PC3) from the Principal Component Analysis (PCA) of morphometric data of male aedeagus (A and B) and of RAD-seq data (C and D).

The analysis of male genitalia revealed strong differentiation of the three Morocco samples, which showed a characteristic *P. tesselatus* aedeagus, as originally described in Drosopoulos & Quartau (2002): with the upper appendages longer and weakly curved, extending beyond the lateral appendages and the lower appendages longer and more regularly curved than *P. spumariu*s (Fig. S1). All the remaining samples showed *P. spumarius*-like aedeagi (Fig. S1). Morphometric analysis confirmed this distinction, with the segregation of Moroccan samples along the first component in the PCA (Figs. 3A and 3B). The variables most associated with this distinction are the mean lengths of upper and of lower appendages of the aedeagus (PCA loadings in Table S2A; boxplots in Fig. S2). The longer appendages in *P. tesselatus* are expected to be related to the longer body size in general in this species (Drosopoulos & Quartau, 2002) but, when controlling for the total length of the aedeagus, the relative size of the lower appendages remained larger in Moroccan samples (Fig. S2).

Within *P. spumarius*, we also noted variation across samples, mainly due to the morphometric variables of length and curvature of the upper appendages (Fig. S2; PCA loadings in Table S2B). In particular, there was some geographical structure, for example UK and Finland lying on one extreme of PC2 and Greece lying on the other, corresponding also to the extremes of latitude in this study. A less accentuated curvature and also smaller length of the upper appendages in the Finnish and British than in the Greek samples may be behind this differentiation (boxplots in Fig. S2).

### Mitochondrial DNA

The fragment of COI spanned 540 bp and was analyzed for 48 specimens, revealing 25 haplotypes, 21 of which had already been described in Rodrigues et al. (2014). The remaining four (haplotypes UK15, UK18, GR18_13 and FIN9) differed from previously known haplotypes by 1 or 2 substitutions (Fig. S3). Two of these new haplotypes (GR18_13 and FIN9) lay in an intermediate position in the haplotype network between the previously defined “North-Eastern” and “Western” haplogroups. In fact, the three haplogroups are not completely distinct but we maintain their designation in order to more easily describe and visualize the mitochondrial variation in relation to the RAD-seq variation: “Eastern-Mediterranean” (EM) in red, “Western” (W) in green and “North-Eastern” (NE) in blue (Fig. S3). We also attributed similar colors to the groups resulting from RAD-seq for ease of visualization.

All seven specimens from Morocco (MOR) sequenced for mtDNA either showed the most common haplotype of the “W” haplogroup (H29) or a haplotype differing by only one substitution (H28 and H37) (Fig. S3). All haplotypes from the Azores (AZO) and continental Portugal (POR) belonged to “W”. France (FRAN) haplotypes belonged in “W” or in between “W” and “EM” (haplotype H49). Haplotypes from Greece (GRE) belonged to “EM” or in between “W” and “NE” (haplotype GR18_13). Haplotypes from Turkey (TUR) belonged to “NE”. In Finland (FIN), there were haplotypes from “EM”, “NE” and also one between “W” and “NE”. The USA population comprised haplotypes from “NE” and the UK population from “NE” and “W” (Fig. S3). The four new haplotypes were submitted to GenBank under accession numbers MT025773–MT025776.

### RAD sequencing

A total of 838,730,936 reads was obtained from the Illumina sequencing. The *process_radtags* step in STACKS retained 647,870,180 reads. This corresponds to an average of 3,811,001 ± 3,524,799 (standard deviation) reads per individual. Thirty-seven individuals with lower numbers of reads (< 500,000 reads) or large amounts of missing data (> 60%) were excluded from the analysis (Table S1), leaving a total of 133 individuals, for which the number of reads ranged from 736,248 to 23,798,148 (average 4,507,853 ± 3,668,879 sd). Raw reads after demultiplexing were deposited in SRA database with accession PRJNA606428. The *population* STACKS module, followed by filtering, produced 1,691 SNPs, with a mean coverage of 105.5 reads per locus per individual (Fig. S4) and mean percentage of missing data per individual of 12.3 % (Table S1). We applied relatively stringent filtering criteria to avoid having large amounts of missing data per individual resulting from the large genome size in this species (2.58 Gbases; Rodrigues et al., 2016). This has produced a relatively small number of SNPs but that have a good representation across individuals and that are expected to be scattered across the genome. Since the draft genome is still incomplete and very scattered we were not able to assess this distribution.

### Population structure

**Figure 4:**
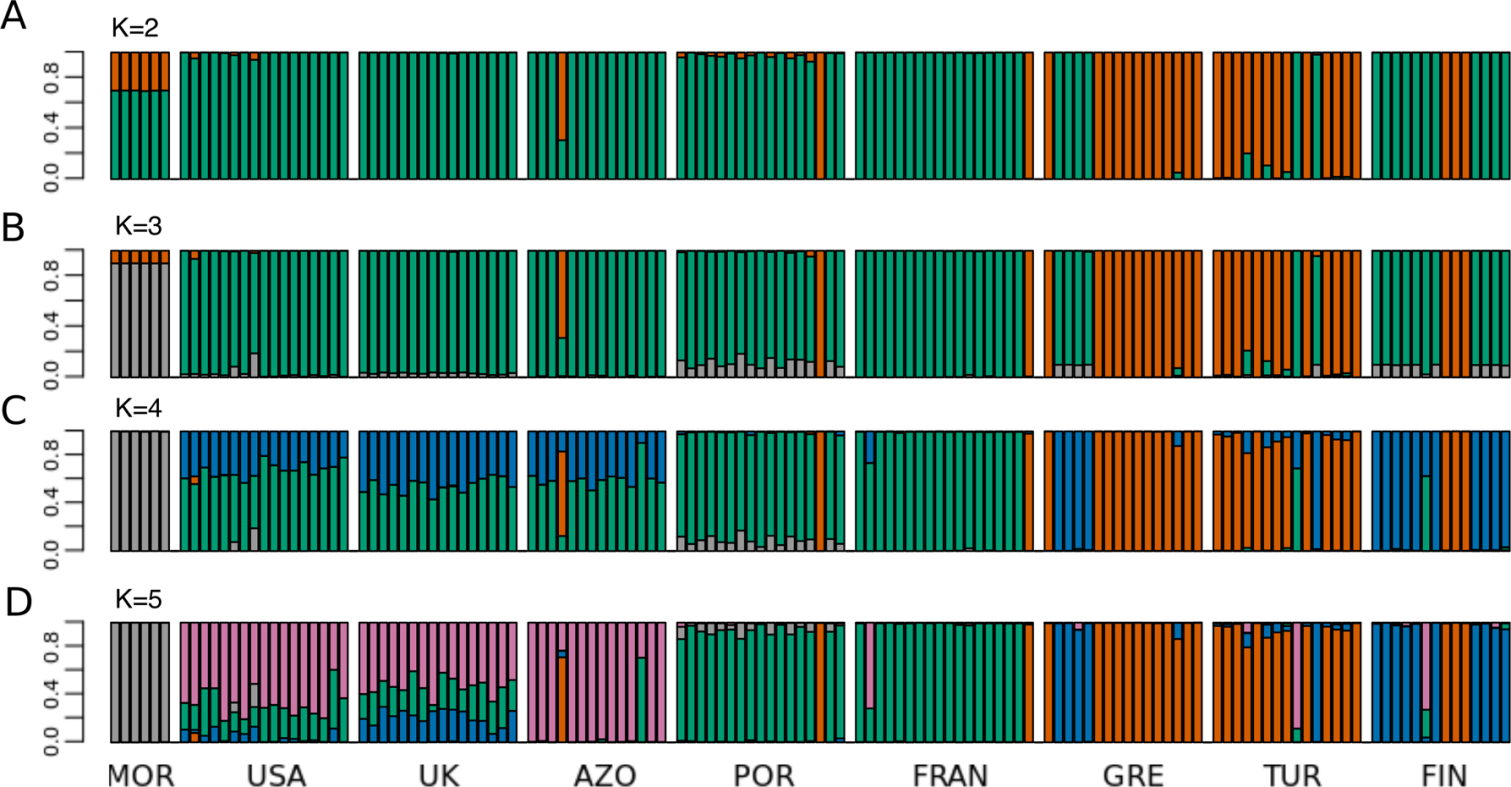
STRUCTURE results for the complete dataset with 9 populations. STRUCTURE results for the dataset including all populations, for (A) K = 2, (B) K = 3, (C) K = 4 and (D) K = 5. The best K according to Evanno, Regnaut & Goudet (2005) method was K = 4. The colors of the major groupings in STRUCTURE were chosen to correspond loosely to the mitochondrial DNA haplogroups (Rodrigues et al. 2014 and this study), for a better visualization.

Principal Component 1 in the PCA clusters Morocco individuals away from the others (Fig. 3C and 3D). When testing other assembly and filter parameters we obtained similar patterns in the groupings of samples (Fig. S5). Also, STRUCTURE analysis gave support to a genetic group solely comprising Moroccan samples (the best K according to Evanno, Regnaut & Goudet (2005) was 4; Fig. 4). The average F_ST_ of Morocco *versus* other populations was 0.4, much higher than average F_ST_ of other populations’ comparisons (0.13) (Table 1). In all population-pairwise F_ST_ calculations involving Morocco, there were a considerable number of SNPs that were fixed or nearly fixed for one allele in Morocco and for the other allele in all the other populations, as seen in the relatively high frequency of high F_ST_ values on the histograms in all comparisons and in the high correlations between F_ST_ values among population pairs (Fig. S6A). There was neither such a high number of fixed SNPs nor such high correlations between F_ST_ values when considering the other pairs of populations (Fig. S6B). Moroccan samples are thus clearly differentiated, at the genome-wide markers, from the remaining eight populations here analyzed in contrast with mtDNA results that showed no differentiation between Morocco and the Iberian Peninsula (Fig. S3).

**Table 1:** Pairwise F_ST_ matrix and estimates of expected and observed heterozygosity (H_E_ and H_O_, respectively) and F_IS_ for each population. The triangular matrix shows the F_ST_ values for each pair of populations and the bottom values are the estimates of expected and observed heterozygosity (H_E_ and H_O_, respectively) and F_IS_ for each population.

	MOR	USA	UK	AZO	POR	FRAN	GRE	TUR	FIN
MOR	0								
USA	0.4557	0							
UK	0.4474	0.0415	0						
AZO	0.4464	0.0785	0.0980	0					
POR	0.3375	0.0755	0.0887	0.1089	0				
FRAN	0.4222	0.0728	0.0925	0.1226	0.0359	0			
GRE	0.4484	0.2050	0.2141	0.1998	0.1831	0.1833	0		
TUR	0.5039	0.2403	0.2503	0.2273	0.2168	0.2018	0.0533	0	
FIN	0.4451	0.0762	0.0964	0.1108	0.1094	0.1188	0.0887	0.1423	0
									
	MOR	USA	UK	AZO	POR	FRAN	GRE	TUR	FIN
H_E_	0.0373	0.0708	0.0737	0.0641	0.0795	0.0725	0.0808	0.0789	0.0781
H_O_	0.0258	0.0460	0.0537	0.0460	0.0406	0.0475	0.0360	0.0491	0.0396
F_IS_	0.3313	0.2927	0.2379	0.2599	0.4421	0.2962	0.4795	0.3544	0.4233

**Figure 5:**
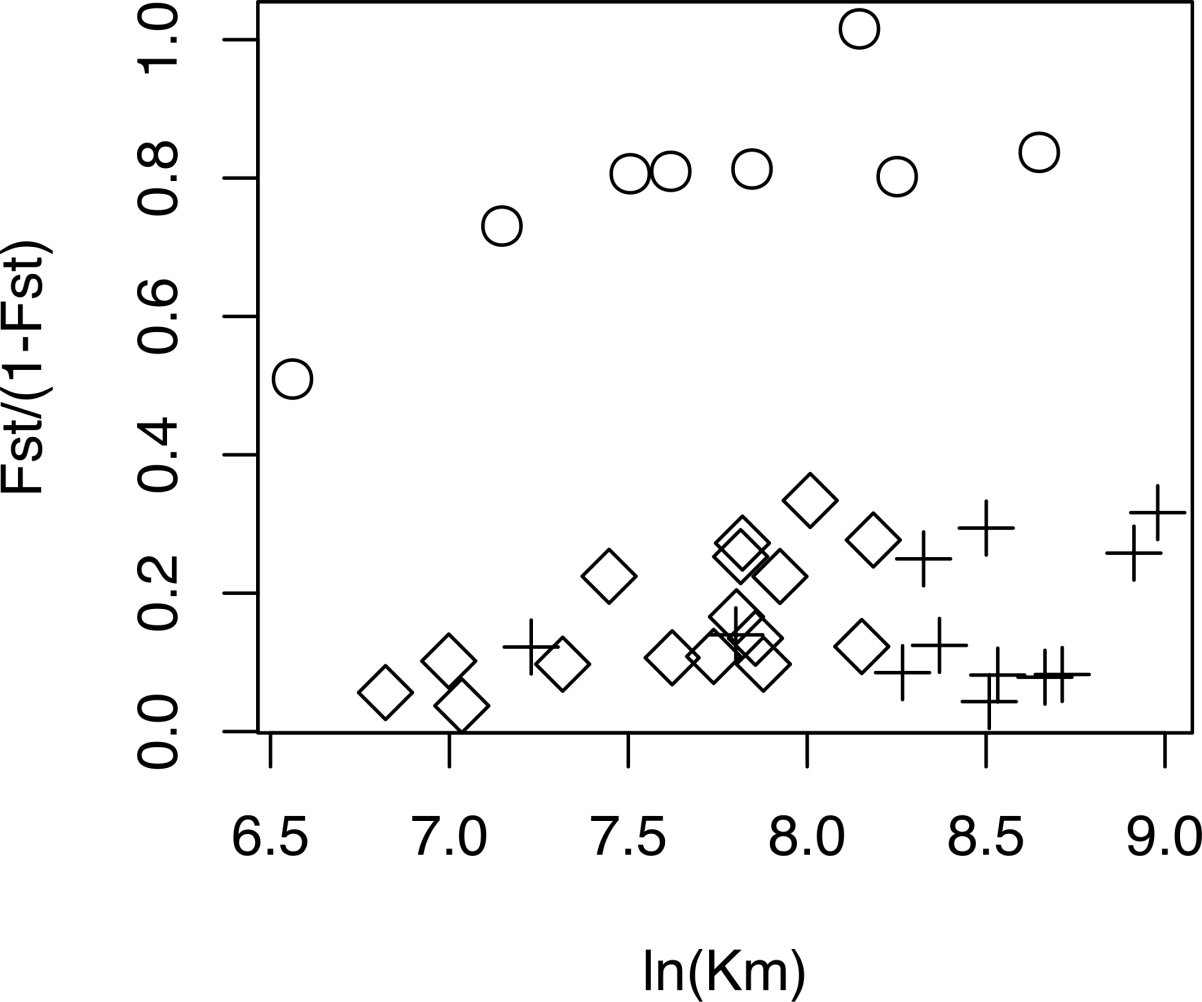
Geographical distance *versus.* genetic distance for each pair of populations. Scatterplot of the geographical distance (natural logarithm) *versus* genetic distance (*F*_ST_∕(1 − *F*_ST_)) for each pair of populations. Symbols discriminate distances between: Morocco and the other populations (circles); European populations (diamond); transatlantic populations (USA or Azores) vs. European populations (crosses).

The relationship between geographical and genetic distances was significant when considering European populations (excluding from the dataset USA, Azores and Morocco) (Mantel test, z =19.49793, p =0.0177; Fig. 5). When considering the comparisons involving Morocco, a positive correlation is seen between genetic and geographical distances, but mainly because of the lower F_ST_ value obtained between Morocco and Portugal (F_ST_ =0.34), than between Morocco and the other populations (F_ST_ > 0.4; Fig. 5). This lower differentiation may be the result of some level of admixture, which was detected in the STRUCTURE analysis, where all individuals from the Portuguese population show a small contribution from the genetic group present in Morocco (Fig. 4).

**Figure 6:**
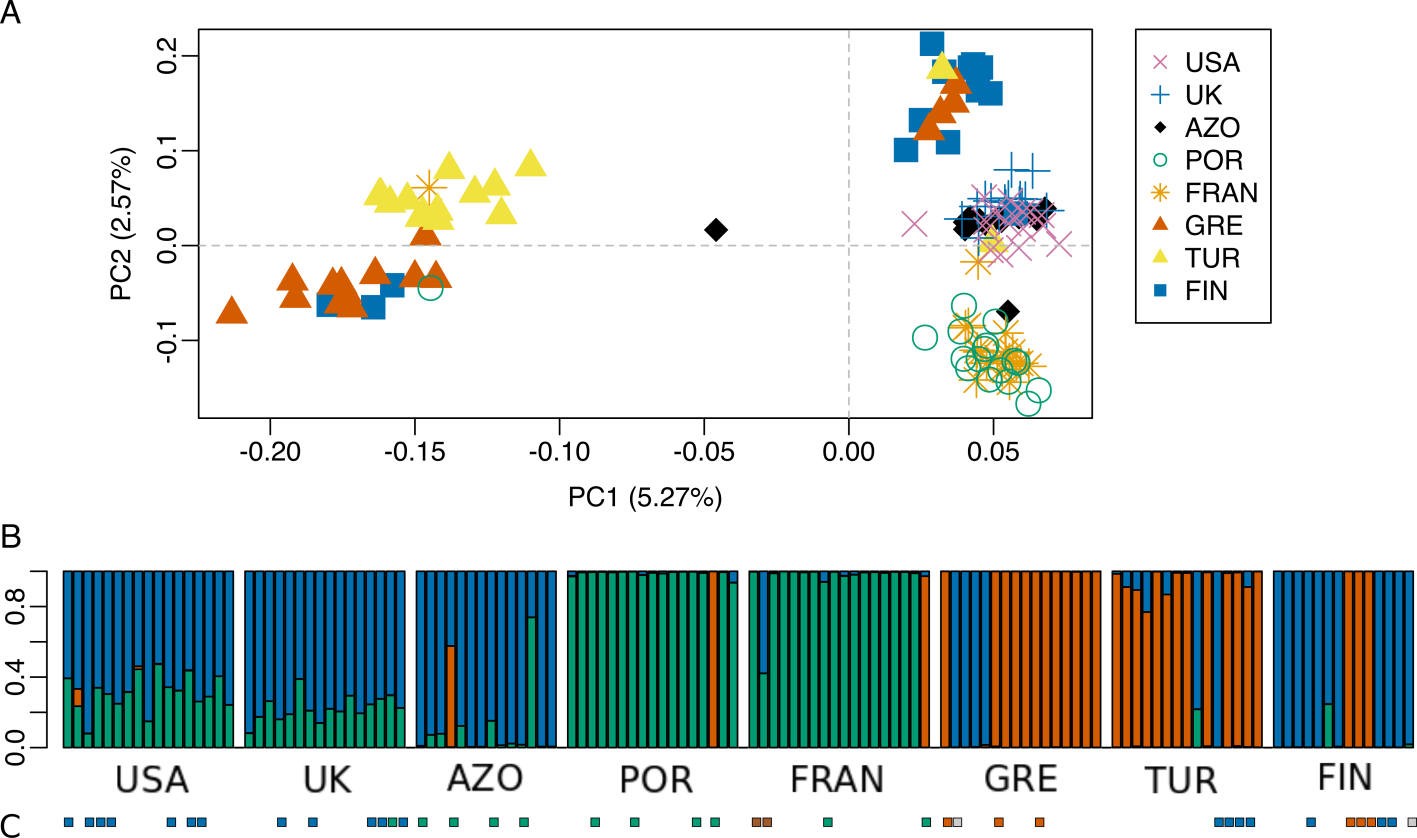
Population genetic structure analysis, excluding the North African population. (A) Scatterplot of the two first principal components (PC1 and PC2) from the Principal Component Analysis of RAD-seq data for the eight populations, after excluding the North African population; (B) STRUCTURE results for K = 3 (best K according to Evanno, Regnaut & Goudet (2005). (C) Mitochondrial haplogroups present in each population (Rodrigues et al., 2014 and this study) shown in colored squares (blue: “North-Eastern”, green: “Western”; red: “Eastern-Mediterranean”). The colors of the major groupings in STRUCTURE were chosen to correspond loosely to the mitochondrial DNA haplogroups, for a better visualization.

Mean diversity (expected heterozygosity, H_E_) ranged from 0.0373 (in Morocco) to 0.0808 (in Greece) and mean observed heterozygosity (H_O_) from 0.0258 (in Morocco) to 0.0537 (UK). H_O_ values were generally lower than expected under Hardy-Weinberg equilibrium (HWE) in all populations (average F_IS_ of 0.346) (Table 1). For Morocco, interpretation of H_E_ should be carried out carefully, since individuals come from three different locations and thus are not necessarily expected to be in HWE. Additionally, we found a positive and significant correlation between observed heterozygosity and sequence read depth (r_S_ =0.686, p =0.0412; Fig. S7). This suggests that lower read depths may have led in some cases to allele dropout, contributing towards false homozygotes. However, in the case of Morocco, the mean read depths were not the lowest in this dataset, being similar to others (Fig. S4) and thus this should not be the main factor contributing to the low observed heterozygosity.

The dataset without Moroccan samples consisted of 127 individuals and 2,083 SNPs. For this dataset, PCA revealed two distinct clusters along PC1, generally separating Greece and Turkey from the remaining populations (Fig. 6A). The latter were separated along PC2 in three groups, one including mainly Portugal and France, another including mainly Finland, and a third one, in between these two, including USA, UK and the Azores (Fig. 6A). This structure had already been detected in PC3 of the analysis of the dataset that included Morocco (Fig. 3D). For this dataset, when excluding the USA and the Azores populations, there was again significant isolation-by-distance for the European populations (Mantel test, z =19.49793, p =0.0229). However, there were a few individuals that were genetically more similar to geographically more distant individuals, which is also seen in the STRUCTURE analysis (Fig. 6). In Greece and Finland, there were no admixed individuals between the two main clusters (“Eastern-Mediterranean” in red, and “North-Eastern” in blue)—they were either from one or the other group, with a few exceptions (Fig. 6B). An analysis of the Turkish population revealed the presence of possibly admixed individuals from these two groups (with a smaller contribution from the “North-Eastern” cluster). In the USA and UK populations, all individuals showed some level of admixture between the “North-Eastern” (blue) and the “Western” (green) clusters. The Azores allies a small contribution from the “Western” group to a major one belonging to the “North-Eastern” group and one individual was admixed between “North-Eastern” and “Eastern-Mediterranean” (also seen in PCA). The admixture in USA, UK and the Azores is also apparent from their intermediate position between the Portugal+France group and the Finland+Greece+Turkey group in the PCA (Fig. 6A). The best K in the STRUCTURE analysis, according to the method by Evanno, Regnaut & Goudet (2005), was 3.

The majority of specimens for which COI sequence was available, had a correspondence between the mtDNA and the genomic cluster. However, there were some specimens showing a mismatch consisting of a mtDNA haplotype belonging in a different genomic cluster (Fig. 6B and 6C; Fig. S3). For example, one individual from UK (UK6) bearing a mtDNA haplotype (H24) belonging to “Western” haplogroup (green), turned up “North-Eastern” (blue) in the genome analysis. Two individuals from France, bearing mtDNA H49 haplotype (intermediate between “Western” and “Eastern-Mediterranean”), came up as differentiated at genomic markers, one “Western” (green) and the other intermediate “Western”/“North-Eastern”. In Greece and Finland, COI sequenced individuals show both a mtDNA and genomic makeup belonging to either “Eastern-Mediterranean” or “North-Eastern”, except two individuals assessed as “North-Eastern” in genomic markers but with a mtDNA haplotype in intermediate position in the network, between “Western” and “North-Eastern” haplogroups. The four individuals from Turkey sequenced for COI belonged in “North-Eastern” haplotypes. While one of them had full ancestry from “North-Eastern” group, the other three had their largest proportion of ancestry from “Eastern-Mediterranean”, based on the genome-wide markers.

For samples for which both morphometric and RAD-seq data was available (*N* = 32), we computed Principal Components Analysis (Fig. S8) and calculated the correlation between PC1 and PC2 scores for both analyses. There was a significant correlation between PC1 from morphometry and PC2 from RAD-seq (r_S_ =0.63, p = 1E-04), while all the remaining were low and non-significant (r_S_ = −0.22, p =0.2194 between PC1 from each; r_S_ =0.1, p =0.5927 between PC2 from each; r_S_ =0.29, p =0.102 between PC2 from morphometry and PC1 from RAD-seq).

### Detection of selection–outlier analysis and environmental associations

Candidate SNPs for positive selection were identified by detection of highly differentiated outliers: eight were detected by BAYESCAN (Fig. S9); and 25 by KLD (quantile 99% KLD 2.037087) in SELESTIM (Fig. S10 ; Table S3). Six outlier SNPs were common to both analyses. No outlier SNPs for balancing selection were detected in the BAYESCAN analysis (Fig. S9).

The BAYPASS analysis detected 163 SNPs associated with environmental variables (BF >15) (Tables S3, S4 and Fig. S11), one of them common to the candidate SNPs detected with Bayescan. Variables showing association were: Longitude, Temperature Annual Range, Precipitation of Driest Quarter, Precipitation of Wettest Quarter, Mean Temperature of Warmest Quarter, Mean Diurnal Range (Mean of monthly (max temp–min temp)). Spearman correlations between these 6 variables were generally low (absolute values below 0.6), with only two values above 0.6 (Table S4C).

When excluding these candidate SNPs (188 in total) from the “full” dataset, creating a “neutral” dataset, the main pattern of structuring was maintained, differing only in admixture proportions at higher values of K (4 and 5) (PCA in Fig. S12 and STRUCTURE in Fig. S13). When analysing the “candidates” dataset, the PCA showed a separation that corresponded generally to longitude variation along PC1 and to latitude along PC2 (Fig. S12). The STRUCTURE analysis, although artificial for the dataset of loci under selection, revealed similar structuring when compared to the other datasets, but with less admixture (Fig. S13). This is an expected outcome considering that these candidate loci have similar allelic variation within each population and different allelic variation between populations. The fact that there are still differentiated individuals within populations in this dataset, consistently assigned to the same groups as in the other datasets, is a reflection of the methods for detecting selection, based on population allelic variation.

Seventy-three candidate SNPs had hits (threshold *e*-value of 1E–30) with the draft genome of *P. spumarius* and seven with the transcriptome (Rodrigues et al., 2016). From these, nine had hits (threshold evalue of 1E–5) with predicted genes in the NCBI nucleotide database (Table S3).

## Discussion

RAD sequencing analysis revealed the genetic distinction of North-African relative to other samples here analyzed, which matched the morphological differences at the male genitalia, identifying these as *Philaenus tesselatus*. This genetic differentiation was however not detected at the mitochondrial DNA level, since *P. tesselatus* and *P. spumarius* share mtDNA haplotypes, as described in previous studies (Maryańska-Nadachowska, Kajtoch & Lachowska, 2011; Rodrigues et al., 2014). These results thus reinforce the importance of taking an integrative approach when studying the taxonomy of a group of species, especially cryptic ones (Edwards & Knowles, 2014; Dejaco et al., 2016; Borges et al., 2017; Tonzo, Papadopoulou & Ortego, 2019).

The fact that there are mitochondrial DNA haplotypes shared between *P. tesselatus* and *P. spumarius*, while the nuclear genome is differentiated, may indicate selection on mtDNA following introgressive hybridization (Gompert et al., 2008). One possible mechanism for selection on mtDNA described in several insects, is the occurrence of maternally inherited endosymbionts, including *Wolbachia*, associated with certain haplotypes. These endosymbionts are known to manipulate reproductive output, mainly through cytoplasmic incompatibility: no viable offspring are produced when an infected male fertilizes an uninfected female, or a female infected with a different strain (Werren, Baldo & Clark, 2008). Since both mitochondria and the symbiont are maternally transmitted, haplotypes associated with the *Wolbachia* infection could thus spread, hitchhiking through the population. Mitochondrial introgression between closely related species caused by *Wolbachia* has been described in several species of Diptera and Lepidoptera (Jiggins, 2003; Narita et al., 2006; Rousset & Solignac, 1995; Whitworth et al., 2007). In these cases, different species share the same mitochondrial haplotypes, making DNA barcoding ineffective. Such a scenario would be interesting to investigate, as *Wolbachia* infection has already been detected in *P. spumarius* across Europe and North America (Lis, Maryańska-Nadachowska & Kajtoch , 2015; Kapantaidaki et al., 2021; Wheeler et al., in press).

The admixture from the Moroccan genetic group detected in all the individuals from the Portuguese population (located in the Central-West part of the Iberian Peninsula) suggests some level of recent or ongoing gene-flow between *P. spumarius* and *P. tesselatus*. Despite previous doubts about the taxonomic status of these two taxa, our data point towards them being closely related but independent gene-pools, probably early in the speciation “continuum” (Seehausen et al., 2014). Both taxa co-occur in some locations in southern Iberian Peninsula (personal observation by JA Quartau and AC Neto, based on identification by male aedeagus morphology) and it will be important to study these sympatric areas. Genital traits are relevant since they may contribute to reproductive isolation, either structural or sensory, if differences in genital morphology between species prevent or reduce the success of copulation and insemination (Masly, 2012). Structural isolation has been shown, for example, in the species pair *Drosophila yakuba* Burla, 1954 and *Drosophila santomea* Lachaise & Harry, 2000 (Kamimura & Mitsumoto, 2012), but in many species no convincing evidence for such isolation has been found so far (Masly, 2012). Morphological variation in female genitalia, as well as behavioural and physiological responses during mating may also aid in understanding potential mechanisms of reproductive isolation, particularly in sympatry. We recognise that species identification based on male genitalia characteristics may be insufficient when there is intraspecific variation with some overlap between species. Although our small *P. tesselatus* samples limit our understanding of the range of its variation, genomic data allow higher resolution in detecting genetic differentiation, but this is not enough to infer species status (Tonzo, Papadopoulou & Ortego, 2019). A more comprehensive study on morphology, mtDNA and genome-wide variation of a wider sample from the Mediterranean region of both *P. spumarius* and *P. tesselatus* is required.

The morphometric geographical variation detected in *P. spumarius* showed some correlation with genetic variation, although the nature of this association was not fully clear. Clinal latitudinal variation, as well as elevation variation, in the shape of male genitalia had already been described in European populations of *P. spumarius* (Wagner, 1955; Wagner, 1959). Shorter and less curved upper appendages were found in the north compared to the south, and in higher than in lower altitudes in the same geographical regions (Wagner, 1955; Wagner, 1959). RAD sequencing data permitted detection of finer population genetic structure within *P. spumarius* than previously known from mtDNA and a limited number of nuclear genes. Although there was a pattern of isolation-by-distance in European populations, there were clear distinctions between groups in the PCA and STRUCTURE analyses not related to geographical distance. The most likely K of 3 in STRUCTURE corresponded loosely to the three mitochondrial haplogroups already described in Rodrigues et al. (2014) and Maryańska-Nadachowska, Kajtoch & Lachowska (2011), but we detected some degree of admixture along contact zones. We found admixed individuals in France, Turkey and Finland, and several other individuals belonging to a different genetic group, with no admixture. This may suggest recent migration or the maintenance of reproductive barriers. In particular, there was almost no admixture between the “Eastern-Mediterranean” and the “North-Eastern” groups. Maryańska-Nadachowska, Kajtoch & Lachowska (2011) described a contact zone in the Carpathians between North-Eastern and South-Western haplogroups (this last group corresponds to our “Western” and “Eastern-Mediterranean” together) and detected heteroplasmic mitochondrial DNA, likely caused by paternal leakage from hybridization between members of these two clades. Interestingly, Lis, Maryańska-Nadachowska & Kajtoch (2015) have found different levels of *Wolbachia* infection between the different mitochondrial lineages of *P. spumarius*. The North-Eastern clade showed a higher proportion of infected individuals than the South-Western. In the Carpathian contact zone, infection was more prevalent in both groups, although they harboured different supergroups of *Wolbachia.* The authors suggest that there may be limited gene-flow between genetically distinct populations through a mechanism of cytoplasmic incompatibility. This could explain the low level of admixture detected in our study between the two genetic groups. A genome-wide survey with a wider sampling of both genetic groups will allow testing these hypotheses.

The intermediate position of UK, USA and Azores individuals in the PCA analysis, as well as the admixture detected in STRUCTURE, suggest they are the result of mixed gene pools. It further corroborates the mtDNA results of Rodrigues et al. (2014) which showed the occurrence of mixed mitochondrial lineages in the UK and USA and that Azores and some USA samples were genetically similar to those from the UK. Across North America, variation in the morphology of male aedeagus in *P. spumarius* was reported by Hamilton (1979) and different mtDNA haplogroups were detected by Rodrigues et al. (2014), leading to the suggestion of multiple colonization events. The analyzed population from New Hampshire (USA) showed very low genome-wide differentiation from the UK population (mean F_ST_ =0.042) compared to other pairwise comparisons in this study, and also a large number of COI haplotypes belonging to the “North-Eastern” haplogroup (5 in the 7 samples analyzed for mtDNA). This supports a likely origin of the North American *P. spumarius* from Northern Europe, perhaps with multiple colonization events and with large effective population sizes. In S. Miguel Island (Azores), only two COI haplotypes, differing by one substitution, have been found so far (in 6 samples, Rodrigues et al., 2014 and this study), which are closely related to the UK haplotypes from the “Western” haplogroup. From the genomic results, this population was more differentiated from UK and USA (mean F_ST_ =0.098 between AZO and UK, and mean F_ST_ =0.078 between AZO and USA) than these two were from one another (mean F_ST_ =0.042 between UK and USA), showing the lowest genetic diversity (expected heterozygosity) of all *P. spumarius* populations here analyzed. These results suggest a likely origin of the Azores colonization from Northern Europe, and that this colonization is likely to have involved a bottleneck event leading to reduced genetic diversity. The low number of color morphs found in S. Miguel (Borges et al., 2018) in this highly polymorphic species further supports this hypothesis, although selective processes may also be involved. Expanding the sampling and analyses will allow more precise determination of the origin, mode (whether or not mediated by man) and eventually the timing of these transatlantic colonization events.

Population genomics approaches provide genome-wide information that is expected to reflect a baseline of neutral processes and, at the same time, allow detection of loci with signatures of selection, deviating from this baseline (Hohenlohe, Phillips & Cresko, 2010). We focused on detecting local adaptation, by finding those loci that are more differentiated (F_ST_) between populations than expected from the neutral background, and also by finding loci that have allelic variation correlated with environmental variation. When discarding such candidate loci for selection from our dataset, the population structure patterns remained very similar to the neutral dataset, which means that these 9% of loci are not affecting the genome-wide neutral pattern of population structure. Despite the usefulness of RAD sequencing for detection of selection in natural populations (Catchen et al., 2017), this analysis is limited by the number of SNPs analyzed and also by the fact that RAD tags are usually distributed non-uniformly across the genome (Lowry et al., 2016). The large genome size of *P. spumarius* makes it more difficult to have a good genomic representation with these scattered markers. Also, genetic signatures of selection in individual loci can be weak and not easily detected in cases of soft selective sweeps (adaptation from standing genetic variation), epistatic interactions among loci or genotype-by-environment interactions (Hohenlohe, Phillips & Cresko, 2010). Whole-genome analyses, by analyzing patterns of diversity, differentiation and linkage disequilibrium along the genome, will be essential to better understand the evolutionary forces of selection and recombination shaping genomic variation (Ellegren, 2014). The fact that we did not detect loci under balancing selection in the BAYESCAN analysis may also be related to low marker density. *P. spumarius* is particularly known for its balanced polymorphism for dorsal color forms and the assessment of population variation in the color-associated loci (Rodrigues et al., 2016) remains to be done.

In the environmental association analysis, the associated variables were primarily longitude and those related to the extreme values and range variation in temperature and precipitation. This analysis is tentative, since we have a low number of populations from a wide geographic range. The low number of hits of the candidate loci with the *P. spumarius* transcriptome may indicate their location was mostly in non-coding regions, while the low number of hits with the partial genome denotes its incompleteness (Rodrigues et al., 2016). A more complete draft genome is now available (Biello et al., 2020), and new genomic and transcriptomic resources will be soon generated for *P. spumarius* which will provide important tools to further explore the molecular basis of adaptation in this species.

Understanding species divergence and the population genetic structure of *P. spumarius* and related species of the genus *Philaenus* is relevant to the management of the eventual progression of the plant pathogenic bacterium *X. fastidiosa*, since their dispersal patterns might aid or constrain disease transmission. Also, the ecological characteristics of different taxa or local populations may be different, for example in host plant preference, ease of acquisition of *X. fastidiosa* or transmission efficiency. Understanding the specific ecology of the vectors has been shown to be crucial in the management of *X. fastidiosa* diseases in America (Redak et al., 2004). Integrating this information is important for epidemiological models of *X. fastidiosa* in Europe and other Mediterranean countries. The risk of *X. fastidiosa* transmission and disease progression is generally expected to be related to long-range human-assisted movements of infected plants and with shorter-range natural dispersal by vectors (EFSA et al , 2019). Genetic studies of *P. spumarius* have shown that it does not constitute a panmictic population and geographical distance is not the only factor restricting gene flow. Other factors have to be taken into account, including habitat fragmentation, barriers to gene exchange such as endosymbionts or behavioural differences, rapid climate changes that may cause major shifts in distribution ranges, as well as unpredictable adaptive responses (Kellermann & Heerwaarden, 2019). Even without detectable gene flow, adults of *P. spumarius* may be able to migrate occasionally, or consistently but without reproductive outcome, and spread the bacterium. More ecological studies on the abundance and distribution of this insect vector through the seasons and across years are needed to understand the dispersal patterns across geographical regions and the potential for disease spread. It will be important to understand the dispersal patterns from South Italy, a potential source of contamination by *X. fastidiosa* and particularly its subspecies *pauca*, which includes the strain associated with OQDS. This is especially true as previous work detected haplotypes from distinct haplogroups in Italy. For example, Rodrigues et al. (2014) detected both “Eastern-Mediterranean” and “Western” haplogroups in North and Central Italy, as well as in Sicily, unveiling the pivotal role of that region in the dispersal patterns of *P. spumarius* among the Mediterranean peninsulas.

## Conclusions

In this study, morphological and genomic analysis allowed a more detailed view of the divergence between *P. spumarius* and *P. tesselatus*, as well as of the population structure and adaptation in *P. spumarius.* We found genome-wide divergence between these two species, despite the lack of mitochondrial DNA differentiation between them. The population genomics approach taken here showed admixture but also co-occurrence of non-admixed individuals in contact zones of diverging mitochondrial lineages of *P. spumarius*. The potential role of *Wolbachia* in shaping these patterns of divergence and introgression should be further explored.

The findings on species divergence and population structure described here point to the need for elucidating the dispersal and ecological requirements of the different taxa and local populations of these vectors for a better management of *X. fastidiosa* progression.

##  Supplemental Information

10.7717/peerj.11425/supp-1 Supplemental Information 1 Supplemental Information

10.7717/peerj.11425/supp-2 Supplemental Information 2 Supplemental Tables

10.7717/peerj.11425/supp-3 Supplemental Information 3 Aedeagus measurements for each individual - raw data Each row shows aedeagus measurements for one individual. TotLen - total length of aedeagus; MidMeanLen - mean length of middle appendages; LowMeanLen - mean length of lower appendages; UpMeanLen - mean length of upper appendages; UpMeanCurv - mean curvature of upper appendages.

10.7717/peerj.11425/supp-4 Supplemental Information 4 SNP genotypes in VCF format after filtering for 9 populations - raw data

10.7717/peerj.11425/supp-5 Supplemental Information 5 SNP genotypes in VCF format after filtering for 8 populations - raw data

## Additional Information and Declarations

### Field Study Permissions

The following information was supplied relating to field study approvals (i.e., approving body and any reference numbers): The field sampling was carried out in 2010 and 2011 on publicly accessible lands in margins of roads or in private lands with owners’ permissions. This is a collaborative study involving researchers from several of the countries where the samples were collected. The studied species, *Philaenus spumarius*, is considered a widespread insect species across the Palaearctic and the Nearctic regions and it is not an endangered or protected species.

### DNA Deposition

The following information was supplied regarding the deposition of DNA sequences: The mitochondrial DNA sequences are available at GenBank: MT025773–MT025776; the RAD sequencing reads are available at NCBI SRA: PRJNA606428.

### Data Availability

The following information was supplied regarding data availability: Scripts used are available at https://github.com/seabrasg/popgenom_Philaenus.git.
