# Supplementary material for: Population structure, adaptation and divergence of the meadow spittlebug, Philaenus spumarius (Hemiptera, Aphrophoridae), revealed by genomic and morphological data
Source: PeerJ. 2021 Jun 1;9:e11425. doi: 10.7717/peerj.11425 (PMC8176912; doi:10.7717/peerj.11425)
Supplement: Supplemental Information 1 [file peerj-09-11425-s001.pdf]

Supplemental Information for:

Population structure, adaptation and divergence of the meadow spittlebug, *Philaenus spumarius* (Hemiptera, Aphrophoridae), revealed by genomic and morphological data

Sofia G Seabra[1*]; Ana SB Rodrigues[1]; Sara E Silva[1]; Ana Carina Neto[2]; Francisco Pina-Martins[1]; Eduardo Marabuto[1]; Vinton Thompson[3]; Mike Wilson[4]; Selcuk Yurtsever[5]; Antti Halkka[6]; Maria Teresa Rebelo[2], Paulo AV Borges[7]; José A Quartau[1]; Chris D Jiggins[8]; Octávio S Paulo[1]

Supplementary Information 1 - ***Preparation and measurements of male genitalia***

Each male genitalia were dissected under a stereo-microscope and placed in a KOH 10% (w/v) solution for 24 hours. The aedeagus was then extracted and glycerol was used both as a dissection and storage medium. For imaging, each aedeagus was transferred to a small drop of glycerol on a glass slide and its orientation was adjusted with the help of entomological pins to be observed under an Olympus BX51 microscope. The device was equipped with a The Imaging Source DFK 23U274 industrial colour camera. Micro-Manager 2.4 software was used for image acquisition. Multiple images of each aedeagus in dorsal from the underside view were acquired at multiple focus distances and saved in TIF format. Acquired images were subjected to focus stacking with the "Extended Depth of Field" plugin (Foster, Van de Ville, Berent, Sage, & Unser, 2004), scaled and measured in ImageJ 1.52p (Schindelin et al., 2012).

Nine measurements were taken from the aedeagus images (Figure 2A). Length measurements were taken using the "Line Selection" and "Segmented Line Selection" tools. For curvature measurements (Figure 2B) we used "Line Selection" and "Angle" tools together with "Simple Bisection Tool" plugin version 1 (available at: https://gist.github.com/lacan/35000e0fac120a78caeaf667a4bb493e). Considering an arc of a circumference as an approximation of the shape of the upper appendage, curvature is here defined as the central angle of the inner arc formed by the appendage itself. The determination of this angle was adapted from Feduccia (1993) and is based on two reference points: the tip of the upper appendage (*A*) and the base of the inner arc formed by the upper appendage (*B*). A perpendicular line (*r*) is drawn to bisect $\overline{AB}$ and intersects the inner arc of the appendage ($\overset{\frown}{AB}$) at point *C*. Two perpendicular lines (*s* and *t*) are drawn to bisect $\overline{AC}$ and $\overline{BC}$, respectively, and intersect with each other at point *D*. The ∠*ADB* corresponds to the central angle of $\overset{\frown}{AB}$ and is the measure of curvature.

References

Feduccia, A. (1993). Evidence from claw geometry indicating arboreal habits of *Archaeopteryx*. *Science*, *259*(5096), 790–793.

Foster, B., Van de Ville, D., Berent, J., Sage, D., & Unser, M. (2004). Complex wavelets for extended depth-of-field: a new method for the fusion of multichannel microscopy images. *Microscopy Research and Technique*, *65*(1-2), 33–42. doi: 10.1002/jemt.20092.

Schindelin, J., Arganda-Carreras, I., Frise, E., Kaynig, V., Longair, M., Pietzsch, T., … Cardona, A. (2012). Fiji: an open-source platform for biological-image analysis. *Nature Methods*, *9*(7), 676–682. doi: 10.1038/nmeth.2019.

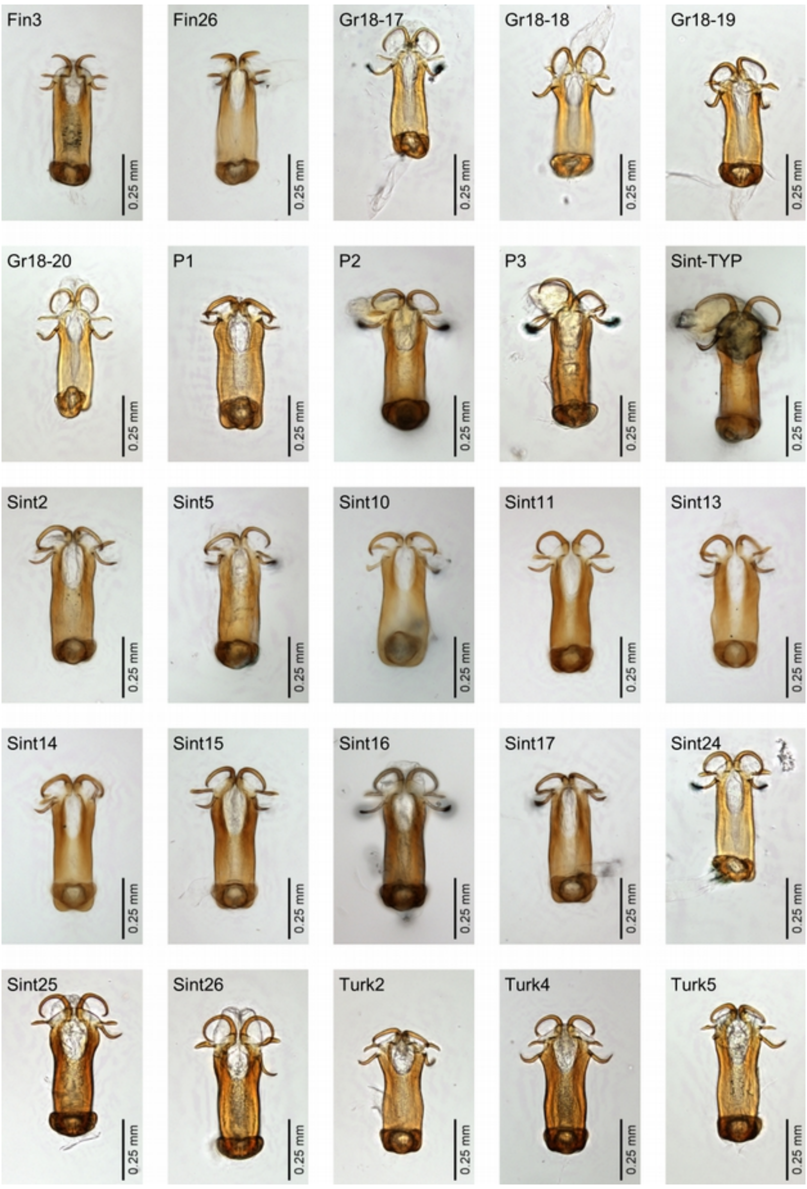

Fin3
Fin26
Gr18-17
Gr18-18
Gr18-19
Gr18-20
P1
P2
P3
Sint-TYP
Sint2
Sint5
Sint10
Sint11
Sint13
Sint14
Sint15
Sint16
Sint17
Sint24
Sint25
Sint26
Turk2
Turk4
Turk5
0.25 mm

(cont.)

(cont.)

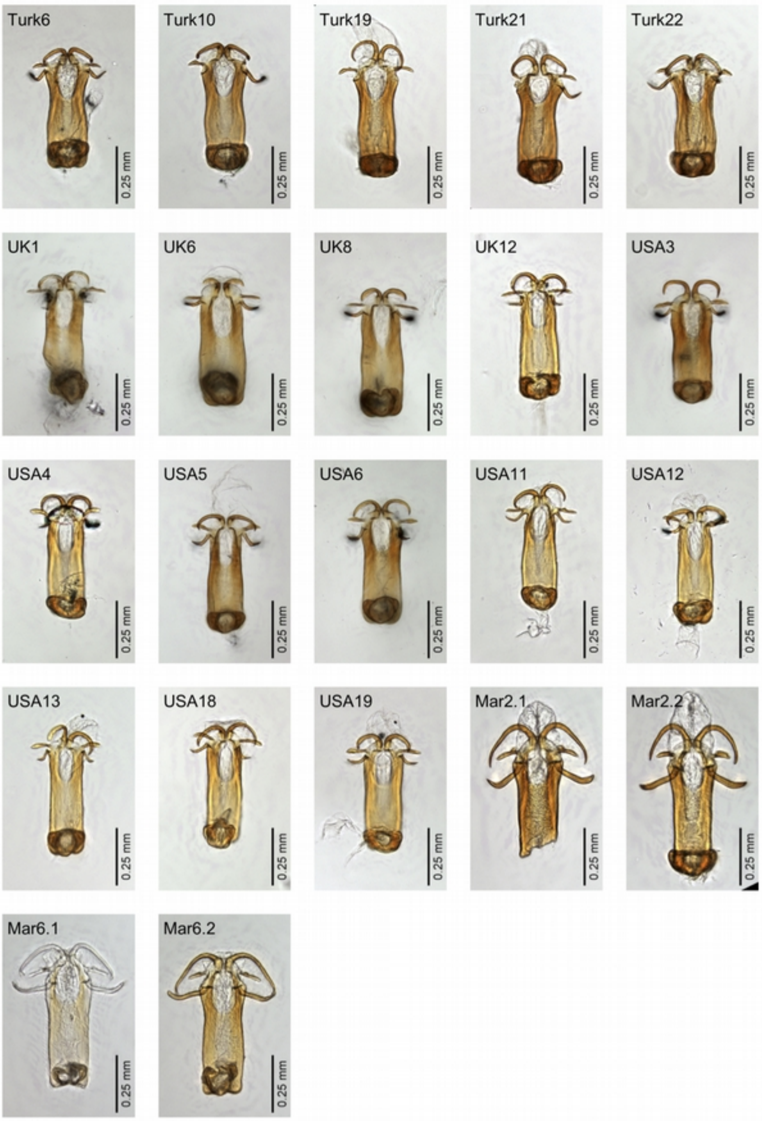

Figure S1- Photographs of each male aedeagus analysed.

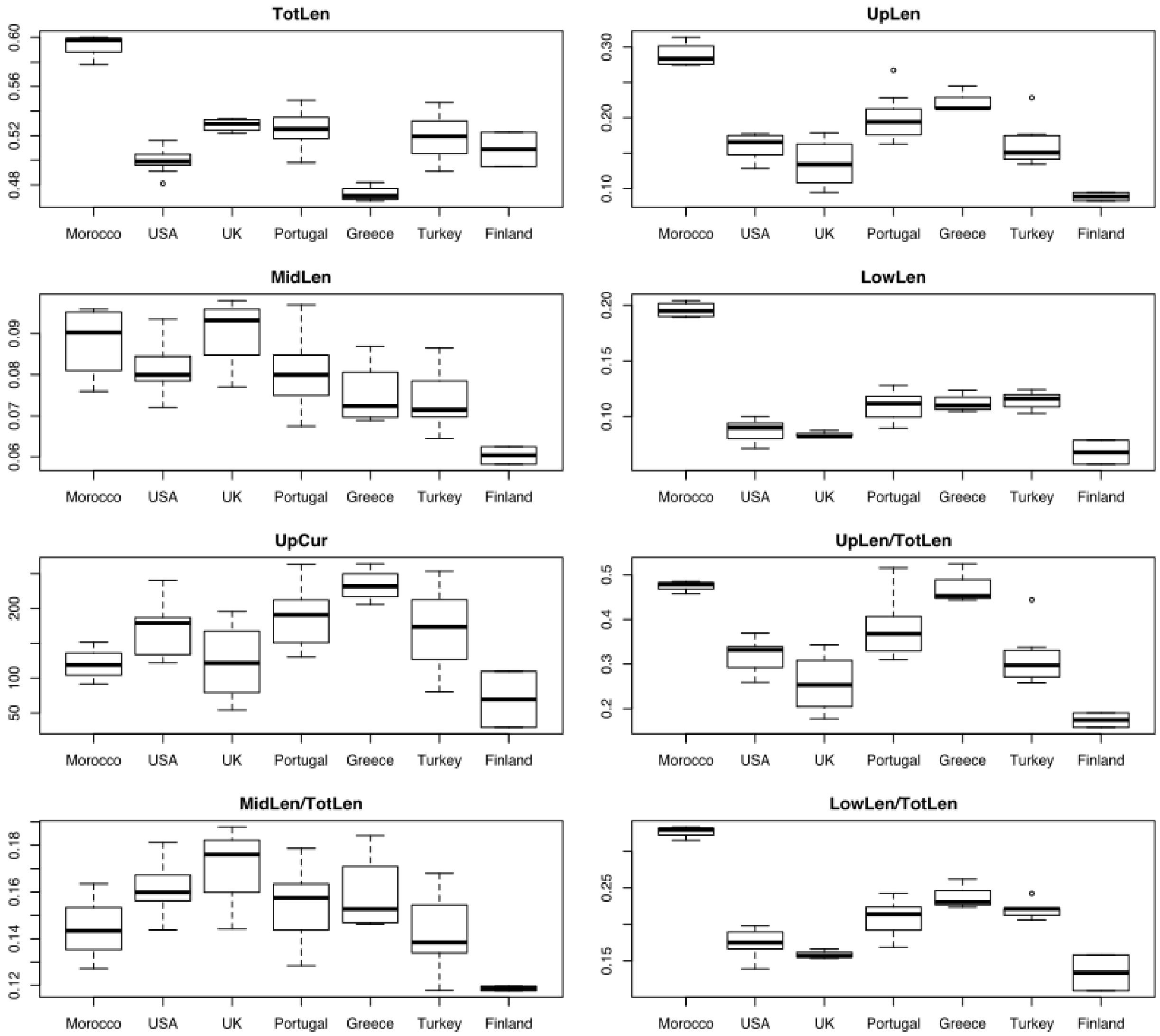

Figure S2 – Boxplots of the aedeagus measurements for each population.

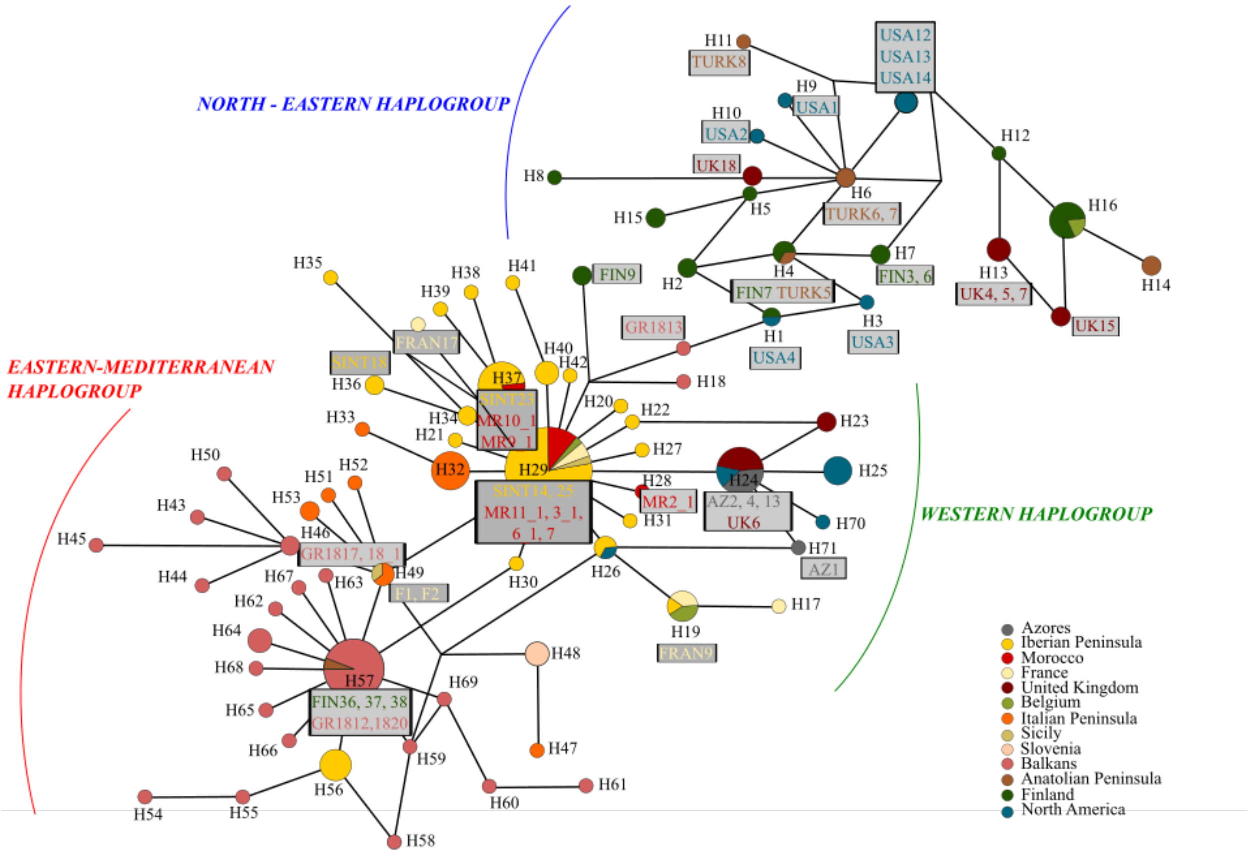

Figure S3 – Mitochondrial DNA (COI) haplotype network (adapted from Rodrigues et al., 2014. CC BY license). Sample names used in the present study are indicated with grey boxes next to the corresponding haplotype.

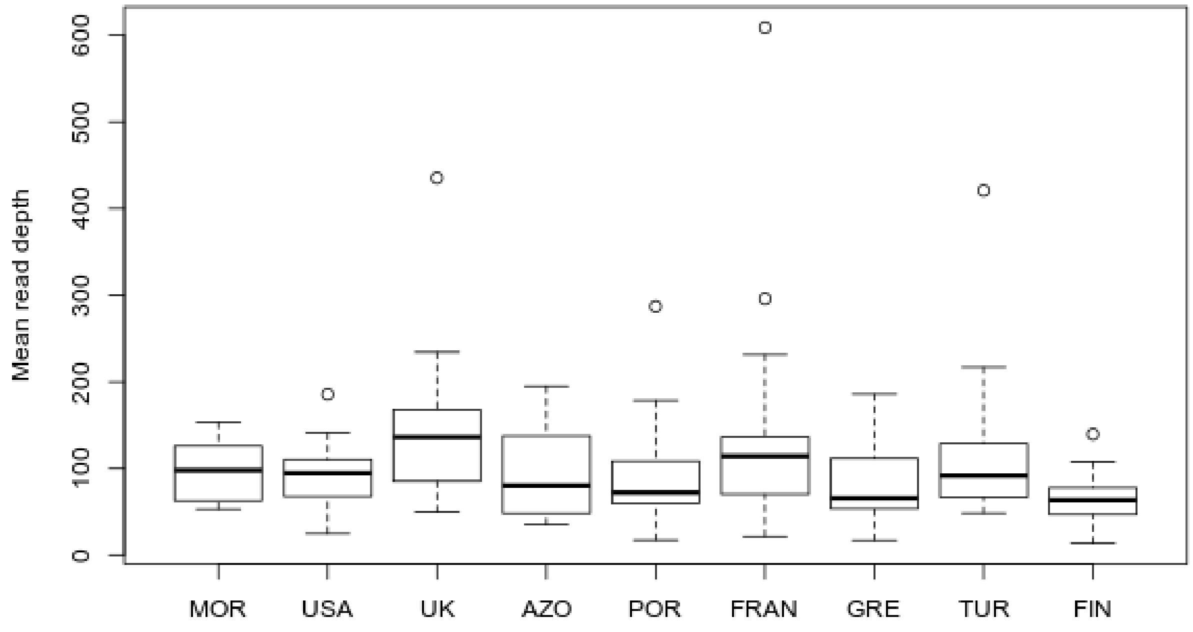

Figure S4 – Mean individual read depth of RAD tags for each population.

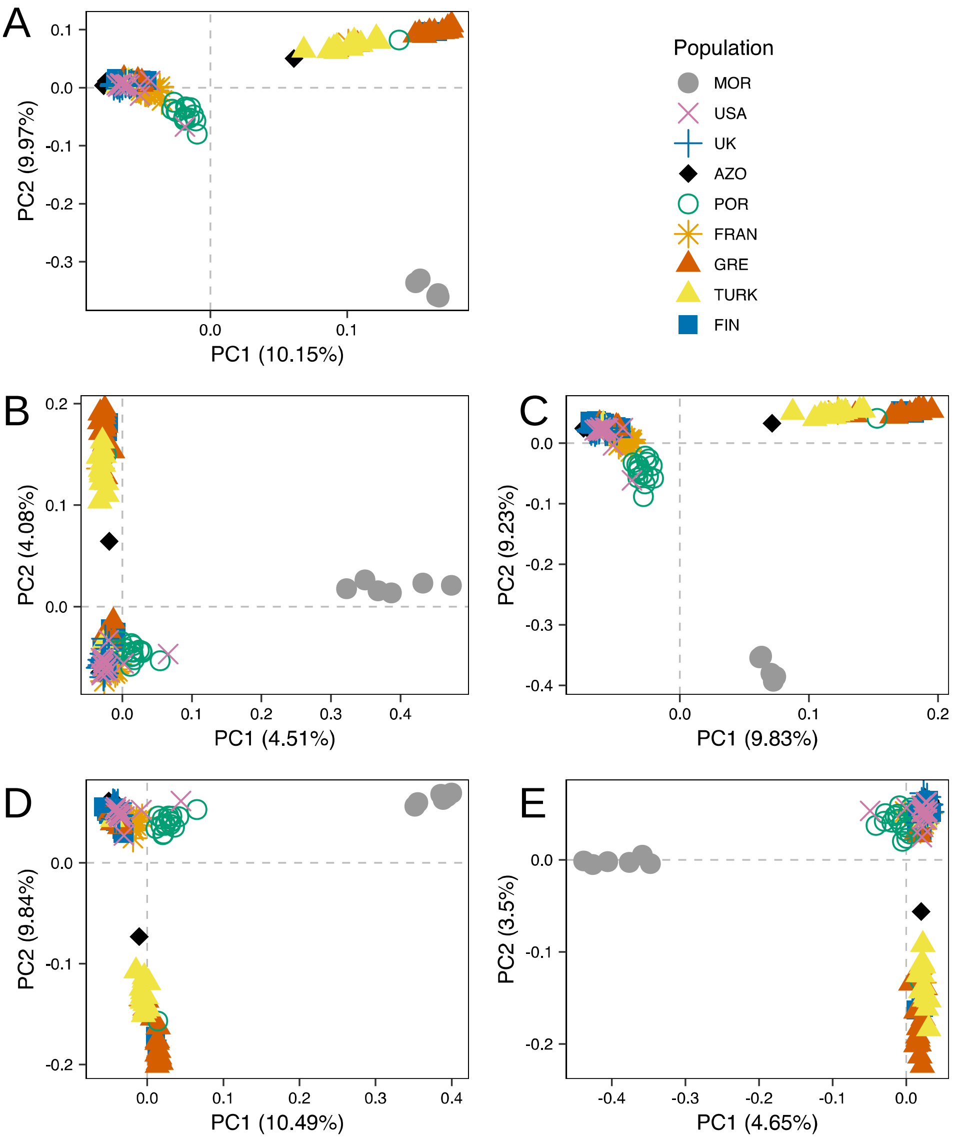

Figure S5 – Principal Components Analysis for datasets with different assembly and filter parameters. A) -M 2, -m 5, -n 4, --min_maf 0.05 (2550 SNPs); B) -M 2, -m 5, -n 1, no min maf (2791 SNPs); C) -M 3, -m 5, -n 4, --min_maf 0.05 (3024 SNPs); D) -M 2, -m 10, -n 4, --min_maf 0.05 (2381 SNPs); E) -M 6, -m 10, -n 1, no min maf (2805 SNPs).

A

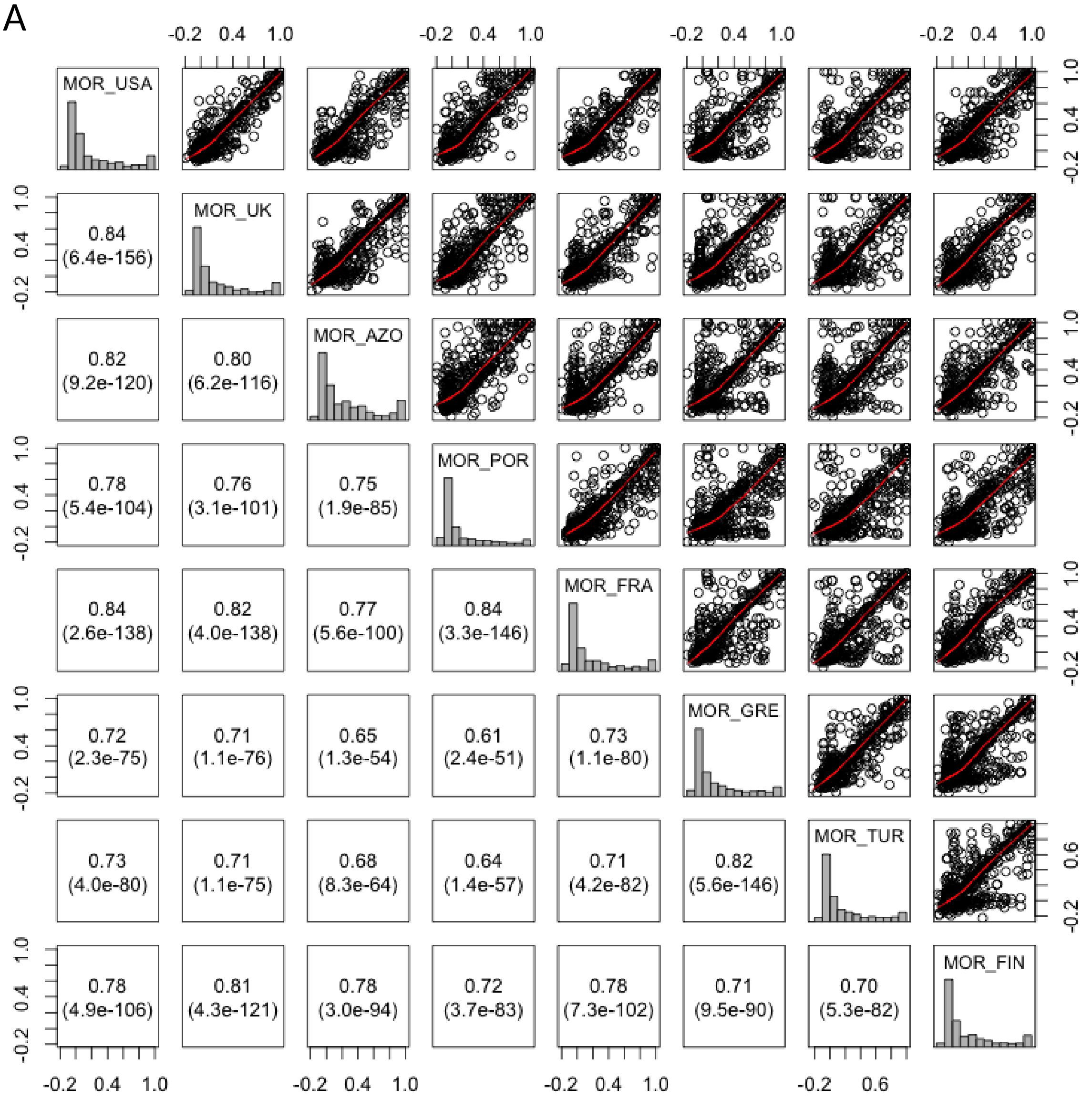
MOR_USA
MOR_UK
MOR_AZO
MOR_POR
MOR_FRA
MOR_GRE
MOR_TUR
MOR_FIN
0.84 (6.4e-156)
0.82 (9.2e-120)
0.80 (6.2e-116)
0.78 (5.4e-104)
0.76 (3.1e-101)
0.75 (1.9e-85)
0.84 (2.6e-138)
0.82 (4.0e-138)
0.77 (5.6e-100)
0.84 (3.3e-146)
0.72 (2.3e-75)
0.71 (1.1e-76)
0.65 (1.3e-54)
0.61 (2.4e-51)
0.73 (1.1e-80)
0.73 (4.0e-80)
0.71 (1.1e-75)
0.68 (8.3e-64)
0.64 (1.4e-57)
0.71 (4.2e-82)
0.82 (5.6e-146)
0.78 (4.9e-106)
0.81 (4.3e-121)
0.78 (3.0e-94)
0.72 (3.7e-83)
0.78 (7.3e-102)
0.71 (9.5e-90)
0.70 (5.3e-82)
-0.2 0.4 1.0
-0.2 0.6

(cont.)

(cont.)

B

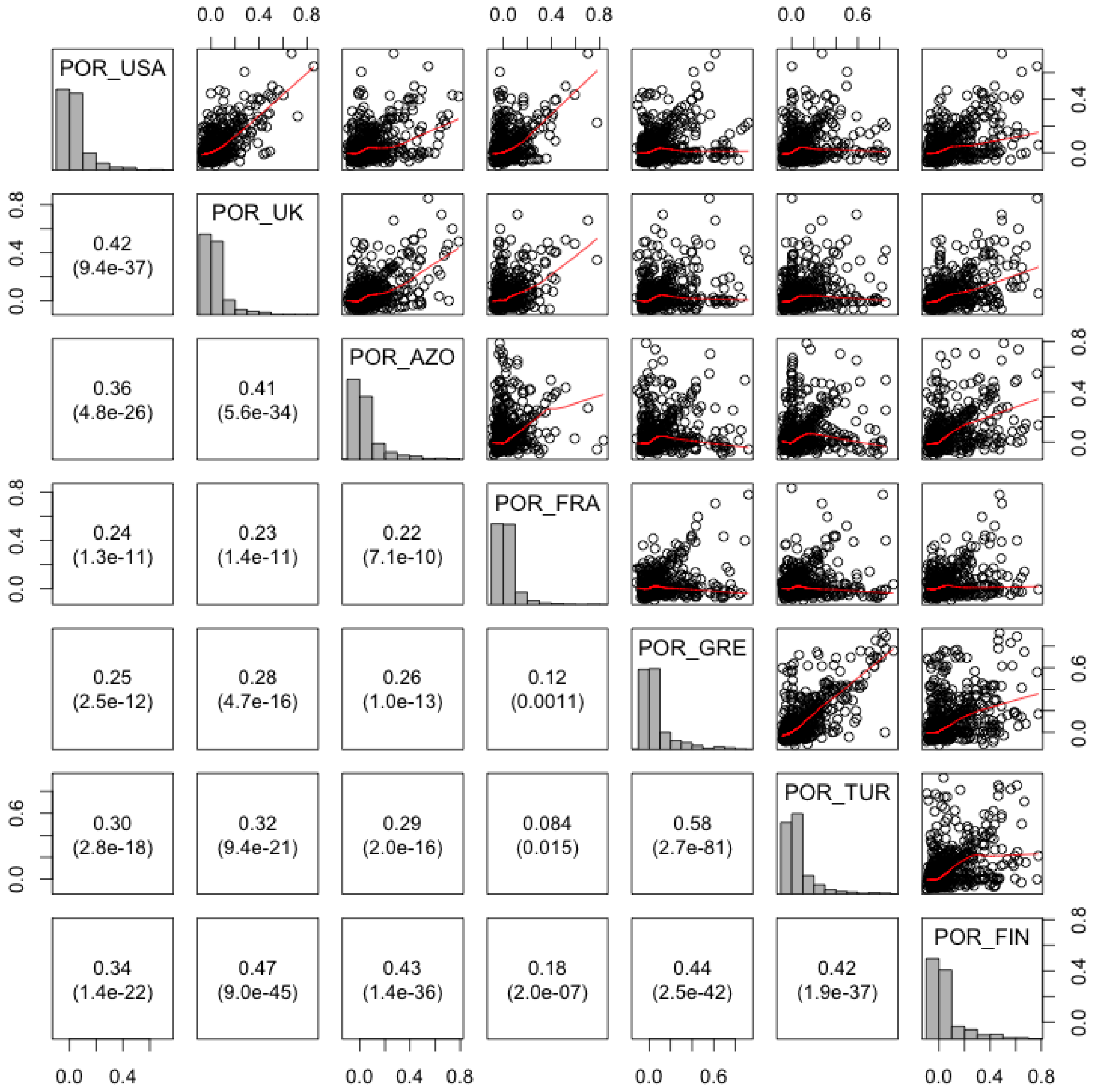

Figure S6 – F_{ST} for each population-pairwise comparison. Diagonal: histograms of FST for the population pair indicated; upper diagonal: scatterplots of F_{ST} for a population pair *vs.* F_{ST} for another population pair; lower diagonal: Spearman correlation estimates (p-values between brackets) for each comparison. A) Comparisons involving Morocco: B) Comparisons involving Portugal.

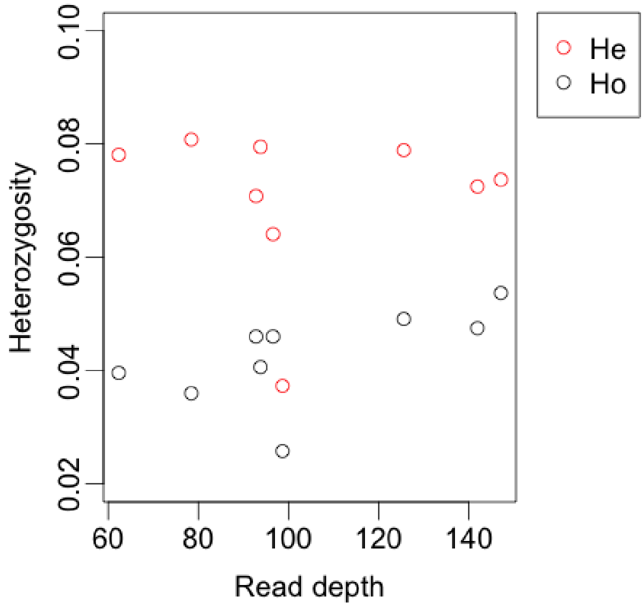

Figure S7 – Mean expected and observed heterozygosity estimates (He and Ho, respectively) *versus* mean read depth of RAD tags for each population.

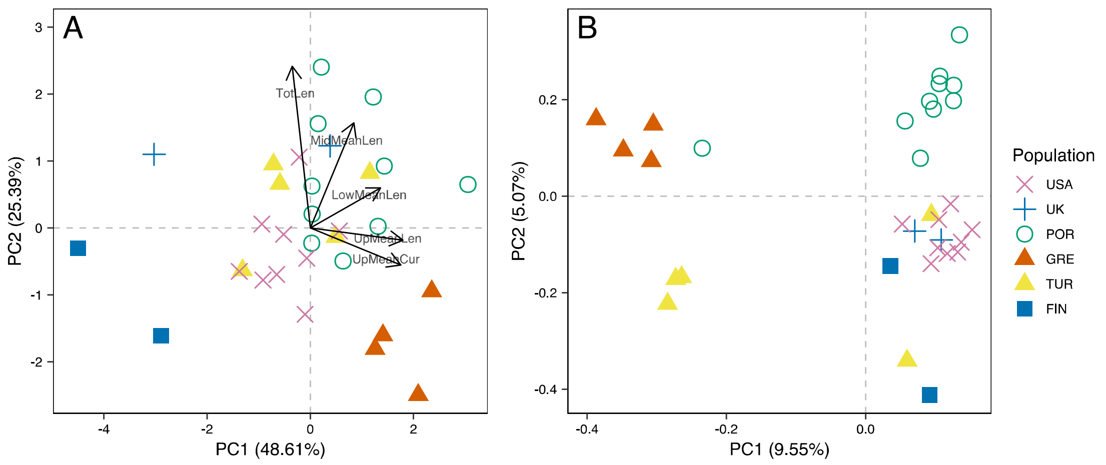

Figure S8 – Principal component analysis (PCA) of morphometric data of male aedeagus (A) and of RAD-seq data (B) for the *P. spumarius* dataset with only the individuals with available data for both analysis (N=32).

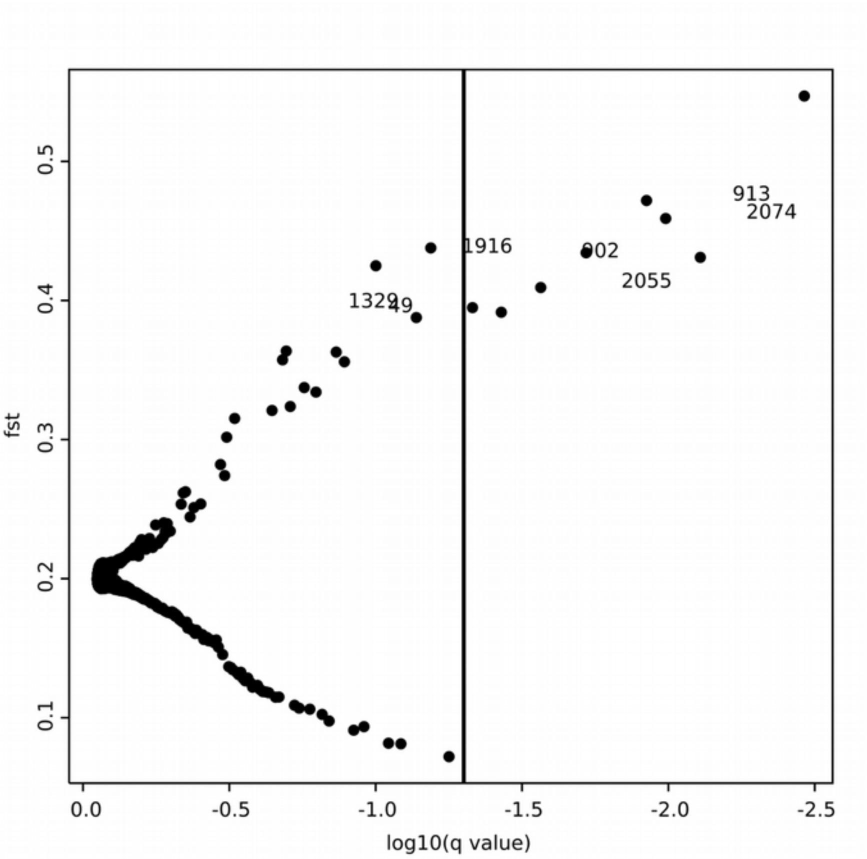

Figure S9 – Bayescan results with significant outliers lying on the right side of the vertical line, corresponding to threshold of 5% of q-values.

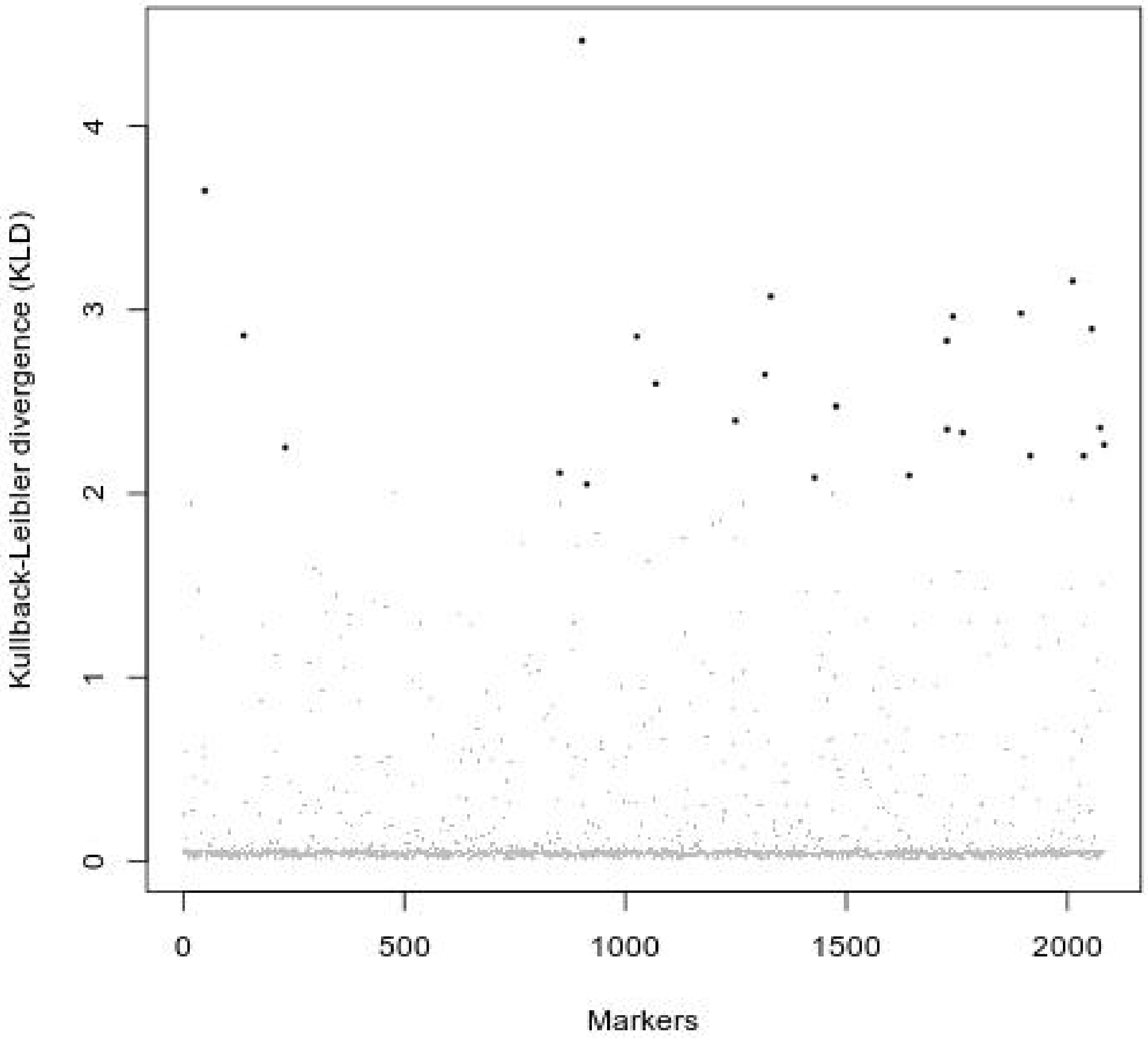

Figure S10 – Selestim results showing the outlier SNPs in black and the remaining SNPs in grey. The threshold limit for the KLD was set to the 99% quantile.

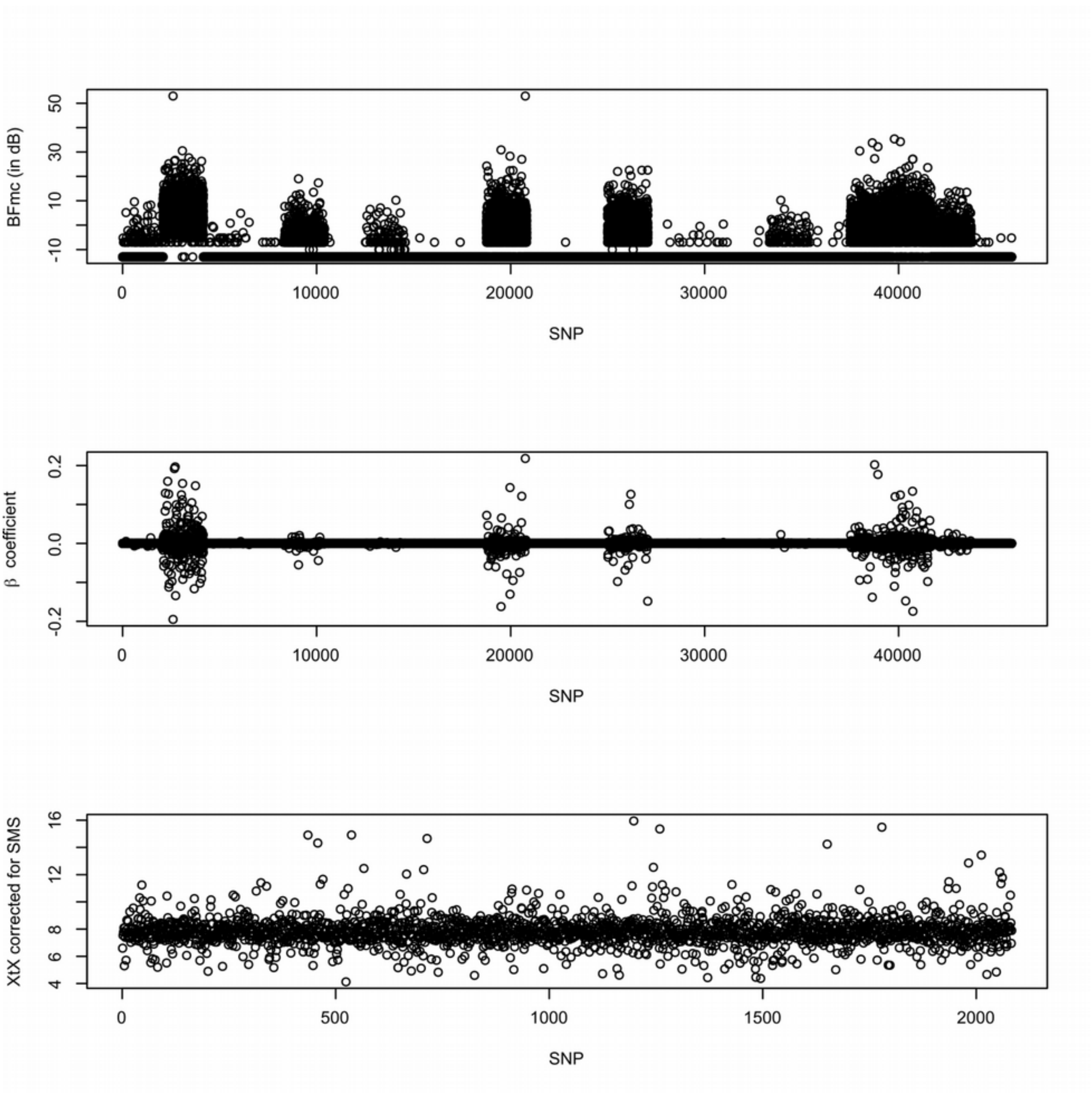

Figure S11- BayPass results

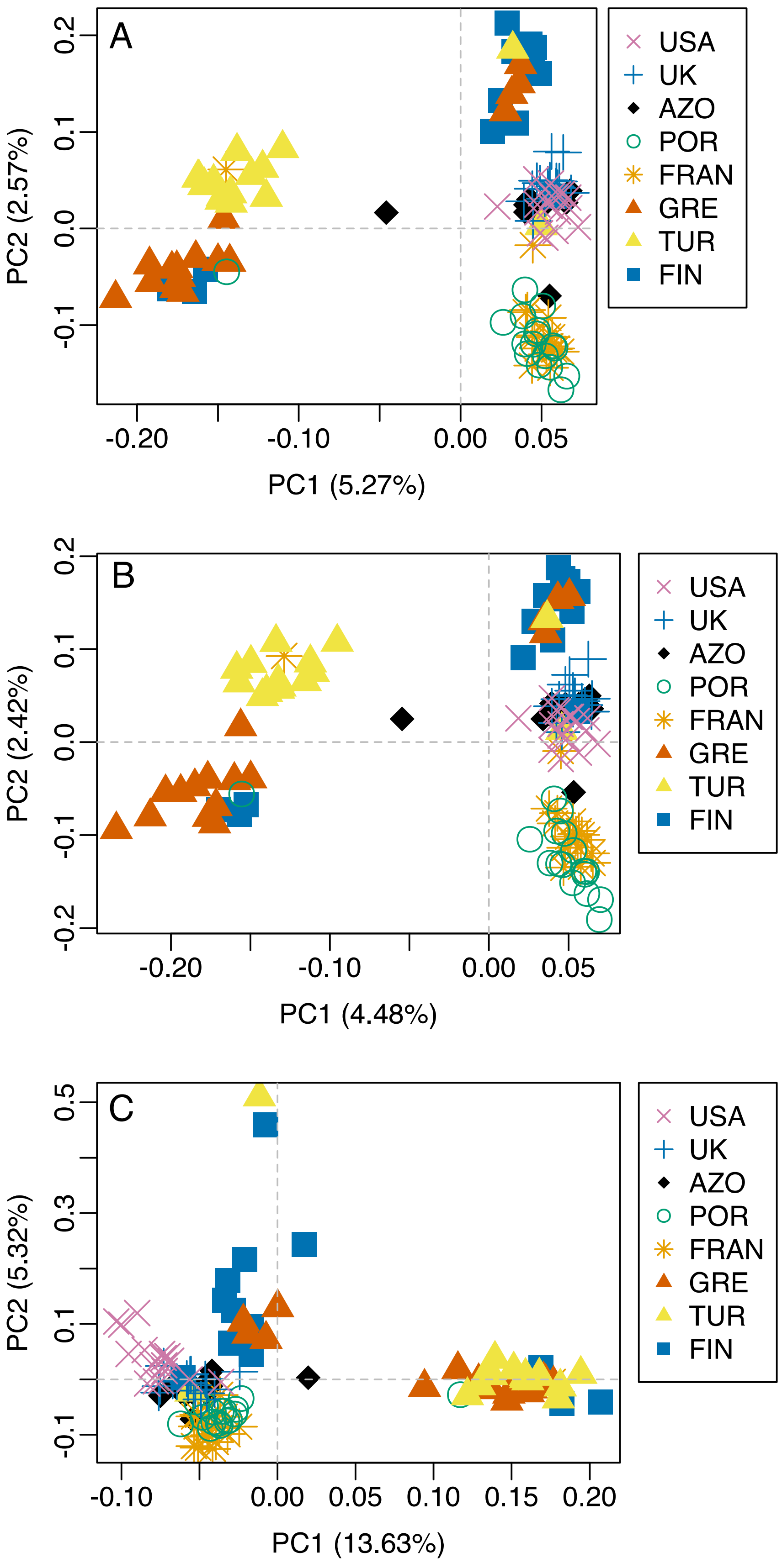

Figure S12 – Principal component analysis (PCA) of RAD-seq data for the datasets: A) Full; B) Neutral; C) Candidate SNPs

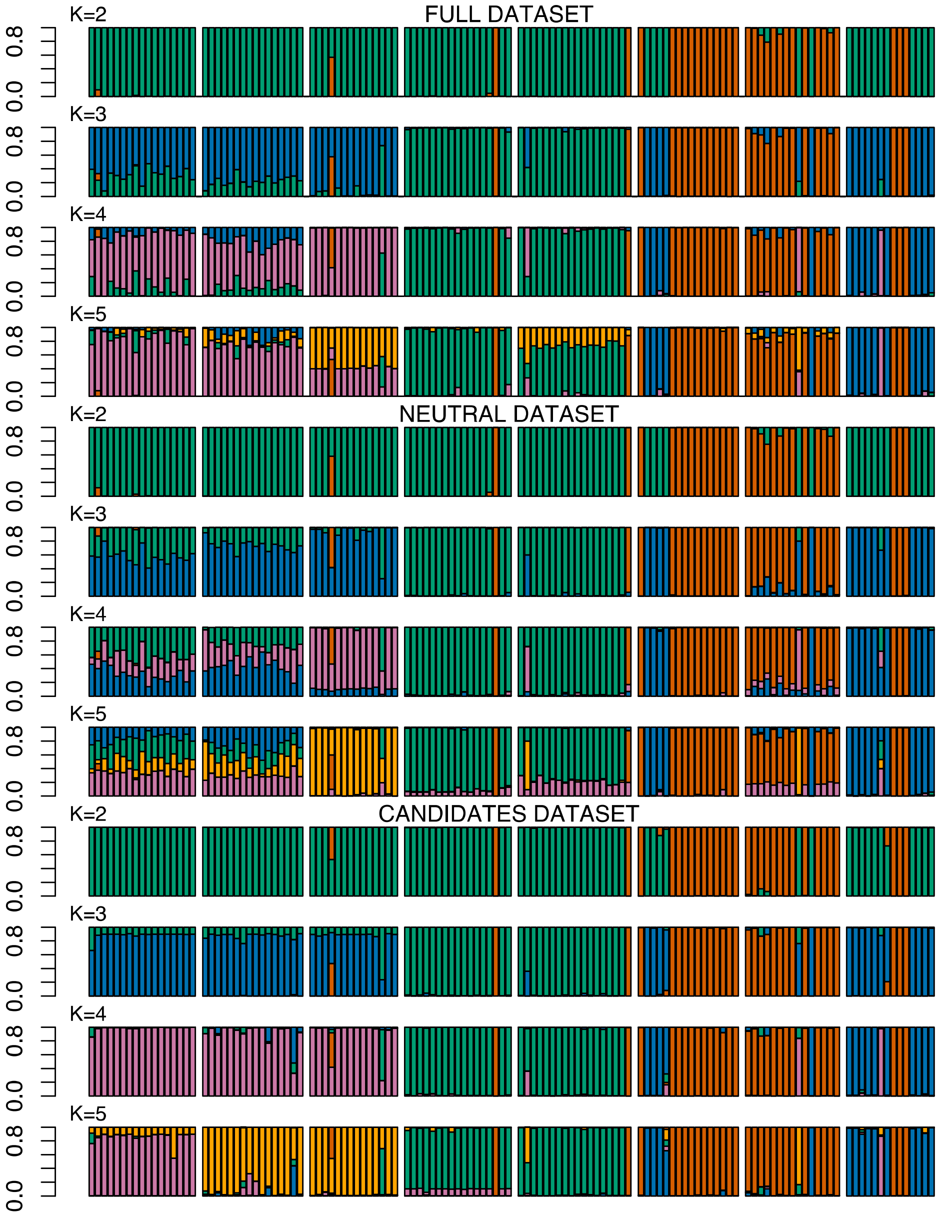

Figure S13 – Structure results of RAD-seq data for the datasets Full, Neutral; and Candidates
